# Cytokinin isopentenyladenine and its glucoside isopentenyladenine‐9G delay leaf senescence through activation of cytokinin‐associated genes

**DOI:** 10.1002/pld3.292

**Published:** 2020-12-21

**Authors:** H. Tucker Hallmark, Aaron M. Rashotte

**Affiliations:** ^1^ Auburn University Auburn AL USA

**Keywords:** cytokinin, cytokinin‐N‐Conjugates, isopentenyladenine, isopentenyladenine‐9‐glucoside, senescence, transcriptome

## Abstract

Cytokinins (CKs) are well‐known as a class of phytohormones capable of delaying senescence in detached leaves. However, CKs are often treated as a monolithic group of compounds even though dozens of CK species are present in plants with varied degrees of reported biological activity. One specific type of CK, isopentenyladenine base (iP), has been demonstrated as having roles in delaying leaf senescence, inhibition of root growth, and promoting shoot regeneration. However, its N‐glucosides isopentenyladenine‐7‐ and ‐9‐glucoside (iP7G, iP9G) have remained understudied and thought of as inactive cytokinins for several decades, despite their relatively high concentrations in plants such as the model species *Arabidopsis thaliana*. Here we show that iP and one of its glucosides, iP9G, are capable of delaying senescence in leaves, though the glucosides having little to no activity in other bioassays. Additionally, we performed the first transcriptomic study of iP‐delayed cotyledon senescence which shows that iP is capable of upregulating photosynthetic genes and downregulating catabolic genes in detached cotyledons. Transcriptomic analysis also shows iP9G has limited effects on gene expression, but that the few affected genes are CK‐related and are similar to those seen from iP effects during senescence as seen for the type‐A response regulator ARR6. These findings suggest that iP9G functions as an active CK during senescence.

## 1 INTRODUCTION

Cytokinins (CKs) are a class of phytohormones involved in a wide range of plant processes (Kieber & Schaller, 2014; Mok & Mok, 2001; Sakakibara, 2006; Skoog & Armstrong, 1970). *Arabidopsis thaliana* (hereafter referred to as Arabidopsis) has been a model species for the examination of CKs and understanding the molecular basis of cytokinin signaling and activity. After biosynthesis and transport, a CK base binds to a CHASE‐domain containing histidine kinase receptor initiating a modified two‐component phosphorelay ultimately resulting in the activation of type‐B Response Regulators, transcription factors (TFs) which mediate the transcriptional response to cytokinin (Hwang et al., 2012; Keshishian & Rashotte, 2015; Kieber & Schaller, 2014; Mok et al., 2000). Despite detailed understanding of the CK signaling pathway, some questions about CK response and activity linger in relation to activity and function of different forms of CK.

In Arabidopsis, detailed CK measurements have revealed nearly 30 different CK forms, with the two most abundant classes being isopentenyladenine‐type (iP) and trans‐Zeatin‐type (tZ) CKs with each class composed of a several metabolites. (Kiba et al., 2013, 2019; Ko et al., 2014; Nam et al., 2012; Nishiyama et al., 2011; Sakakibara et al., 2005; Svačinová et al., 2012; Tokunaga et al., 2012; Werner et al., 2010; Zhang et al., 2014). The iP‐type CKs detected in Arabidopsis include the following: iP riboside 5ʹmonophosphates (iPRP), the first product in the CK biosynthetic pathway and a precursor to active CK; iP riboside (iPR), generally considered a transported form of iP with some bioactivity; iP (base form of CK), capable of binding CK receptors and initiating CK signaling; and iP‐7‐ and iP‐9‐glucoside (iP7G, iP9G), composed of iP bases irreversibly conjugated to a glucose molecule by UGT76C2/1 and generally considered permanently inactivated (Hou et al., 2004; Kieber & Schaller, 2014; Mok et al., 2000; Wang et al., 2011). Both iP7G and iP9G are N‐glucosides, meaning the glucose molecule is attached via a nitrogen atom; these differ from O‐glucosides which occur in tZ‐type CKs and can be hydrolyzed back to tZ bases. Based on previous CK measurement data in Arabidopsis, iP7G is the most abundant iP‐type CK, and is often the most abundant of all CKs (Hošek et al., 2020; Kiba et al., 2019; Nishiyama et al., 2011; Sakakibara et al., 2005; Šimura et al., 2018; Svačinová et al., 2012; Tokunaga et al., 2012). Although iP9G is far less abundant than iP7G, it is often present at appreciable levels, and significantly more concentrated than other metabolites such as iP and iPR (Hošek et al., 2020; Kiba et al., 2013, 2019; Ko et al., 2014; Nishiyama et al., 2011; Šimura et al., 2018; Tokunaga et al., 2012).

Despite the high abundance of iP7G and iP9G, collectively referred to as iPNGs, little is known about the activity of these compounds. Work from the 1980’s demonstrated that Cytokinin‐N‐glucosides (CKNGs) are metabolically stable and do not appear to be converted into other forms of CK (Deleuze et al., 1972; Palni et al., 1984). One recent study using radiolabeled isotopes confirmed this is the case in Arabidopsis cell cultures (Hošek et al., 2020). Other classes of CK such as tZ also have N‐glucoside forms, and previous work has shown that trans‐Zeatin‐N‐glucosides have bioactivity in some assays, yet show distinct effects on the transcriptome and proteome of Arabidopsis seedlings (Hallmark et al., 2020).

Here we show that exogenous application of iP, as well as iP9G, is capable of delaying chlorophyll degradation in detached cotyledons senescence assays. To investigate the mechanism behind this phenomenon, RNA‐sequencing was performed on cotyledons following a detached leaf senescence CK‐bioassay. Surprisingly only three genes were differentially expressed after iP9G treatment as opposed to more than 10,000 genes from iP treatment. Genes differentially expressed after iP treatment were enriched for roles in photosynthesis, translation, and CK response. Fv/Fm analysis validated that iP treatment leads to higher photosystem II efficiency than mock treatment. Altogether, these data suggest CK compounds should not be labeled binarily as active or inactive; modifications to CK bases, as seen in the formation iPNGs by glucosylation of iP, may simply alter or dampen the function of the molecule as opposed to fully eliminating its activity as previously believed. Additionally, this is the first published senescence transcriptome to include a naturally occurring CK base and its glucosides and reveals potential mechanisms by which iP delays senescence.

## 2 MATERIALS AND METHODS

### 2.1 Plants material and growth conditions

Seeds were sterilized with 70% ethanol and 20% bleach with 0.05% Tween before being plated on full strength MS agar plates. Seeds were then stratified 4 days at 4°C in the dark before being moved to a growth chamber running a 16 hr/8 hr light cycle (100 µE) with diurnal temperature of 22°C/18°C. All plants were of the wildtype Col‐0 ecotype of Arabidopsis. Promoter GUS pARR6::GUS lines and *arr6* mutant lines were previously described in To et al., (2004). The *cyp735A1,2* double mutant was previously described in Kiba et al., (2013).

### 2.2 Cotyledon senescence assay

The cotyledon senescence assays were carried out as described in Hallmark et al., (2020). Briefly, cotyledons of 12 day after germination Arabidopsis seedlings were excised at the petiole and floated on 3 mM MES buffer, pH 5.7. Buffer was supplemented with either 1 or 0.1 µM CK (iP, iP7G, iP9G) or an equivalent volume of DMSO as a solvent control. CK were obtained as analytical standards from OlChemIm (Olomouc, Czech Republic). Previous work with CKNGs obtained from OlChemIm revealed no contamination with CK bases including no conversion of CKNGs to CK base during chlorophyll retention assays (Gajdošová, 2011). Once in solution, cotyledons were placed in the dark at 20°C for six days, after which chlorophyll was extracted and quantified as previously described (Sumanta et al., 2014). Three independent biological replicates were performed with 15 cotyledons per replicate.

### 2.3 Root growth assay

Seedlings were germinated on full strength MS agar. Four days after germination, seedlings of uniform size were transferred to MS plates supplemented with 1 µM CK (iP, iP7G, iP9G) or an equivalent volume DMSO (0.1%). Transferred seedlings continued to grow until day 9, at which point root growth since day 4 was measured using ImageJ (NIH). Three independent biological replicates were performed with at least eight seedlings per replicate.

### 2.4 Shoot regeneration assay

Shoot regeneration was completed similarly to a previously described assay (Pernisova et al., 2018) with modifications. The assay was carried out under standard growth conditions described above 1 µM NAA and 1.5 µM CK (iP, iP7G, iP9G) or an equivalent volume of DMSO (0.15%). Fresh weight of calluses was measured 21 days after hypocotyls were transferred to hormone containing media. Three independent biological replicates were performed with at least 5 calluses per replicate.

### 2.5 Transcriptome and gene expression analysis

Cotyledons (*n* = 20 per replicate) were collected from three biologically independent cotyledon senescence assays performed as described above. RNA was extracted using a Qiagen RNeasy Plant Mini Kit according to the manufacturer's instructions. RNA was sent to Novogene, Inc. for quality check, library preparation, and sequencing on an Illumina HiSeq X. Read quality was evaluated using FastQC and trimming was performed using Trimmomatic (Andrews, 2010; Bolger et al., 2014). Read mapping, quantification, and differential expression analysis were carried out using HISAT2, HTSeq, and DESeq2, respectively (Anders et al., 2015; Love et al., 2014; Pertea et al., 2016). At least 20 million paired‐end reads were generated per sample, and these reads mapped back to the TAIR10 Arabidopsis reference genome with a mapping rate of >97% for every sample (Cheng et al., 2017). The significance threshold for differential expression was set at adjusted *p* < .05. GO analysis was performed using AgriGO 2.0 (Tian et al., 2017). Raw sequencing data is available for download at NCBI Sequence Read Archive under BioProject ID PRJNA616296. qRT‐PCR confirmation of RNAseq was completed using Sybr‐green and sequence‐specific primers as shown in Table S1 using an Eppendorf Realplex2 as previously described (Zwack et al., 2013). Heatmap was generated using Heatmapper (Babicki et al., 2016).

### 2.6 Promoter GUS reporter analysis

PromoterARR6::GUS expressing cotyledons plants were treated as detailed above for the senescence assay. After treatment cotyledons were placed into GUS staining solution as in Glazebrook and Weigel, (2002). Cotyledons were vacuum treated for 20 min to increase GUS solution penetration into tissue, and then placed at 37°C overnight, after which, they were removed from the GUS staining solution and placed in 70% ethanol until the tissue had cleared. Ethanol cleared samples were examination under Nikon SMZ1500 with a 1.0× lens with 1.0× or 2.5× magnification and representative cotyledons (*n* = 10) from three independent biological replicate treatments were photographed under identical conditions.

### 2.7 Chlorophyll fluorescence analysis

Following three biologically independent cotyledon senescence assays performed as described above, cotyledons had Fv/Fm measured using standard settings from FluroCam7 software on a Handy FluorCam (Photon Systems Instruments) as described previously (Zwack et al., 2016). Three independent replicates consisting of ten cotyledons per treatment were performed.

### 2.8 Protein content determination

To measure protein content following cotyledon senescence assays, described above, cotyledons were collected from three independent replicates (*n* = 10 per treatment per replicate). Protein content was determined using a previously described protocol with minor modifications (Li et al., 2017). Cotyledons were ground in protein sample buffer (62.5 mM Tris‐HCl, 2% [w/v] SDS, 10% [v/v] glycerol, 5% beta‐mercaptoethanol, 0.005% [w/v] bromophenol blue). Solutions were then boiled for five minutes and centrifuged for 10 min at 10,000 g. Supernatant was collected and 25 µl per sample was loaded into an Invitrogen 12% NuPAGE Bis‐Tris gel. Electrophoresis was carried out at 115 V for 100 min. Proteins were visualized with Coomassie Brilliant Blue staining, and ImageJ (NIH) was used to quantify staining.

### 2.9 Histochemical staining

For visualization of cell death in tissue following cotyledon senescence assays, described above, cotyledon tissue was stained with Trypan Blue as previously described (Fernández‐Bautista et al., 2016). Briefly, cotyledons were submerged in Trypan Blue stain for 25 min and gently shaken before stain was removed and cotyledons were de‐stained. Three independent biological replicates were performed using ten cotyledons per treatment.

### 2.10 Data and statistical analyses

Unless otherwise stated, physiological data presented is the mean ± SE of at least three independent biological replicates, and statistical significance was determined by Student's two‐tailed *t*‐tests.

## 3 RESULTS

### 3.1 Isopentenyladenine and one of its glucosides delays chlorophyll degradation in detached cotyledons

**FIGURE 1:**
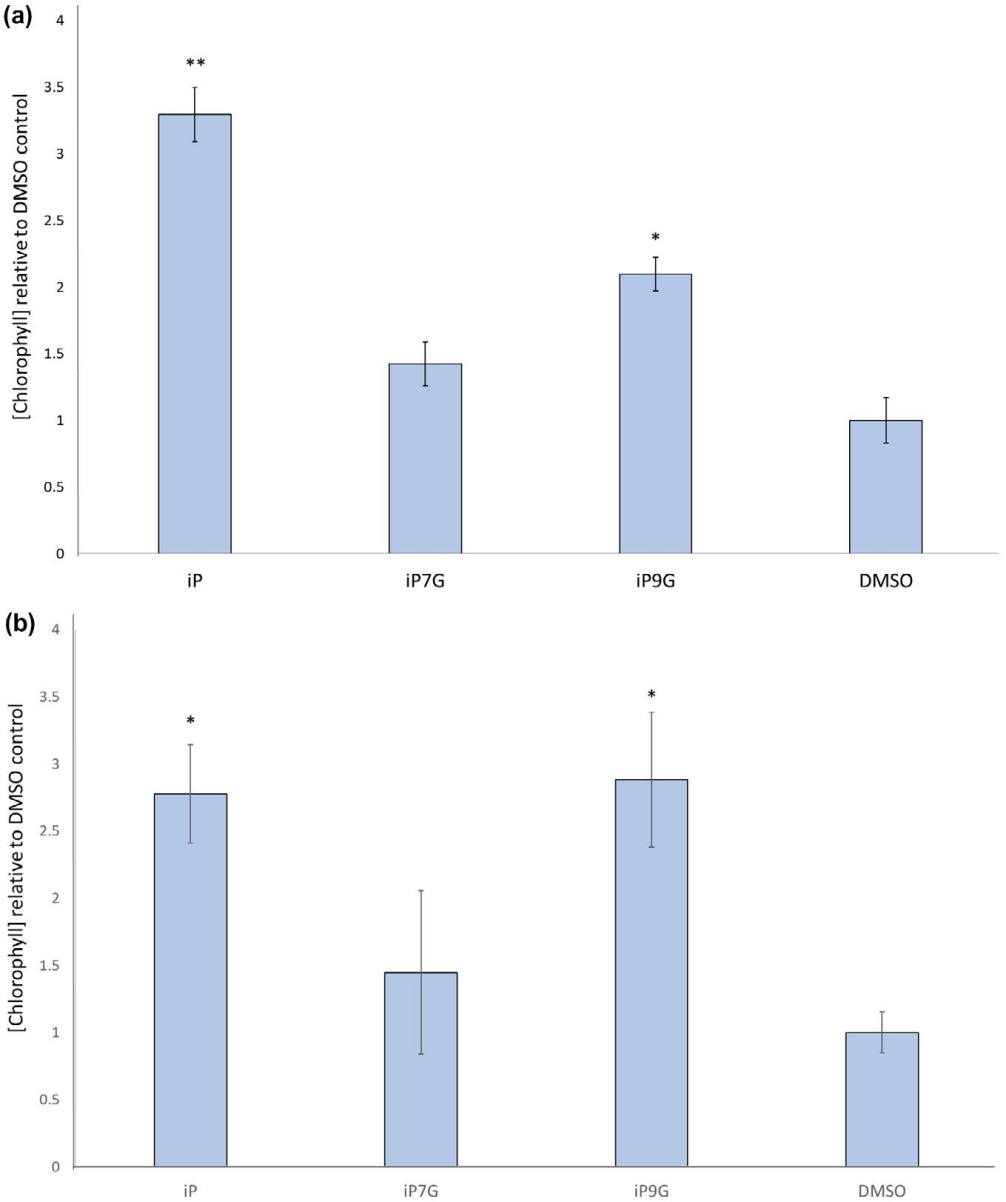
Isopentenyladenine and its 9‐glucoside delays senescence in detached cotyledons. Cotyledons from 12dag seedlings were floated on 3 mM MES buffer supplemented with (a) 1.0 µM or (b) 0.1 µM of indicated hormone or 0.1% DMSO as a solvent control. Average ± SE of three independent replicates. Student's *t*‐test, *p < .05 **p < .01

A hallmark of active CKs is their ability to delay senescence in leaves (Biddington & Thomas, 1978; Gan & Amasino, 1996; Singh et al., 1992; Singh et al., 1992). As the process of chlorophyll degradation is intrinsically linked with leaf senescence, measuring chlorophyll serves as a proxy for measuring how much senescence has occurred (Hörtensteiner, 2006). Here, cotyledons from 12 day after germination (12dag) seedlings were excised and floated on buffer supplemented with 1.0 µM or 0.1 µM CK, either iP, iP7G, or iP9G, or a DMSO control. After six days in the dark, cotyledons treated with iP retain roughly 2–3 times as much chlorophyll as the control at either concentration (Figure 1). iP9G‐treated cotyledons also showed a similar level of chlorophyll retention approximately 2–3 times control levels, while the difference between iP7G and the control at either dose was not significant (Figure 1). The significant reduction in chlorophyll degradation by iP and iP9G treatment suggests both compounds are capable of delaying senescence in detached cotyledon, while iP7G can not. As an additional measure to determine that only iP based compounds are responsible for the delay of senescence seen here, the same assay was performed with an iP treatment of the biosynthetic *cyp735A1,2* double mutant that blocks any conversion of iP to tZ type cytokinins (Figure S1). In the *cyp735A1,2* background iP was able to significantly delay chlorophyll loss similar to that seen in the WT background (Kiba et al., 2013; Figure S1).

### 3.2 Isopentenyladenine‐N‐glucosides do not promote shoot regeneration nor inhibit root growth

**FIGURE 2:**
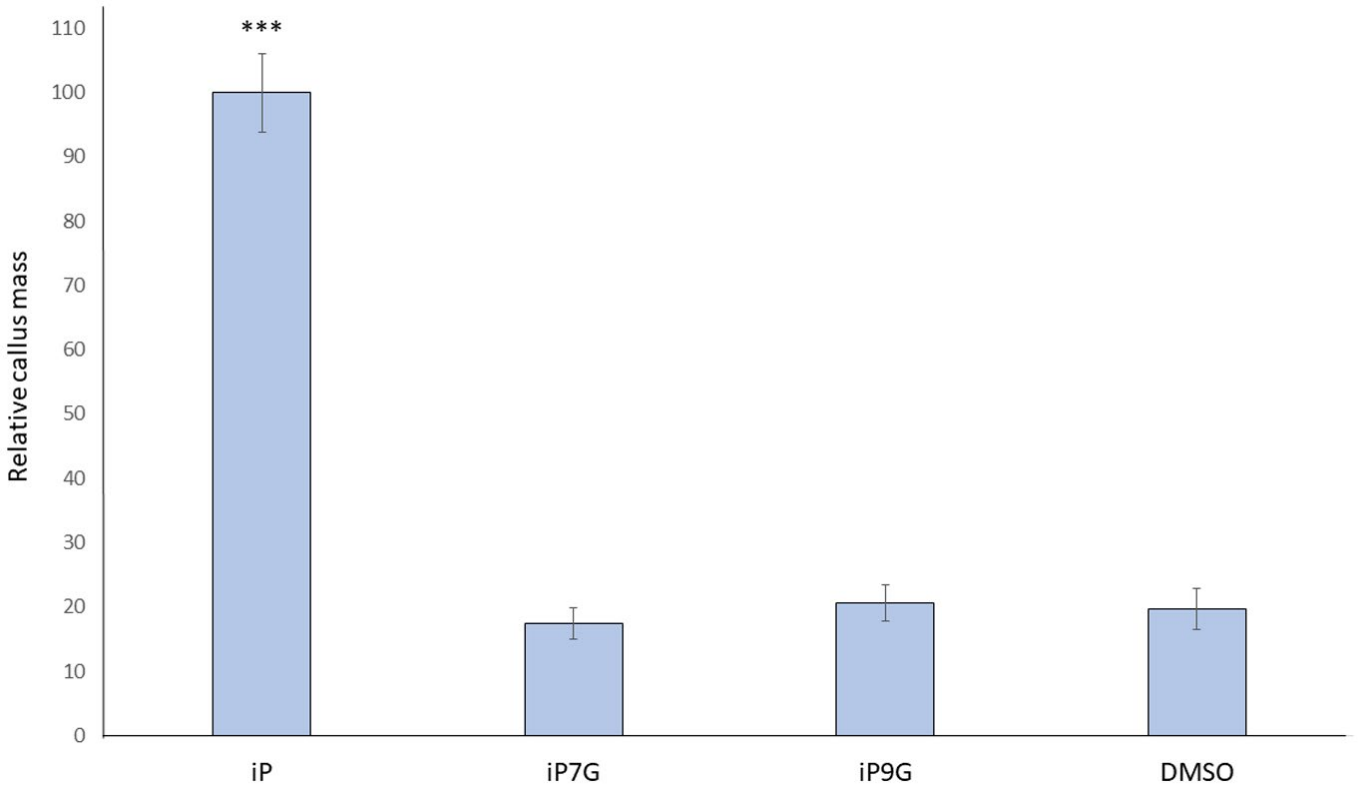
Isopentenyladenine‐N‐glucosides do not promote shoot regeneration. Hypocotyls from 5dag etiolated seedlings were placed on MS + 1% sucrose media supplemented with 1 µM NAA and 1.5 µM of the indicated CK, or an equivalent volume of DMSO as a negative control. Plates were incubated at standard growth conditions for 21d, after which point callus mass was measured. Average ± SE is presented from three biological replicates of at least five calli per replicate. Student's *t*‐test, ***p < .001

Other key features of active CKs include their abilities to promote shoot regeneration in callus and inhibit root growth (Kieber & Schaller, 2014; Mok & Mok, 2001; Skoog & Miller, 1957). To assess whether iPNGs are functionally active as CKs in shoot regeneration, a regeneration assay was performed similarly to previously described protocols (Pernisova et al., 2018). Hypocotyls from etiolated 5dag seedlings were placed on shoot regeneration media (MS containing 1 µM NAA and either 1.5 µM iP, iP7G, or iP9G) and were grown under standard conditions. After three weeks on this new media, callus fresh weight was measured. If the CKs are active, one would expect a significant increase in callus weight, as the combination of active CK and auxin in the media promote shoot regeneration; this results in growth of the callus through reprogramming of the callus tissue to become shoot tissue (Gordon et al., 2007; Pernisova et al., 2018). Calluses on plates containing iPNGs were of similar weight to calluses grown on media containing DMSO control, while callus on iP plates weighed significantly more, indicating only iP was promoting shoot regeneration (Figure 2).

**FIGURE 3:**
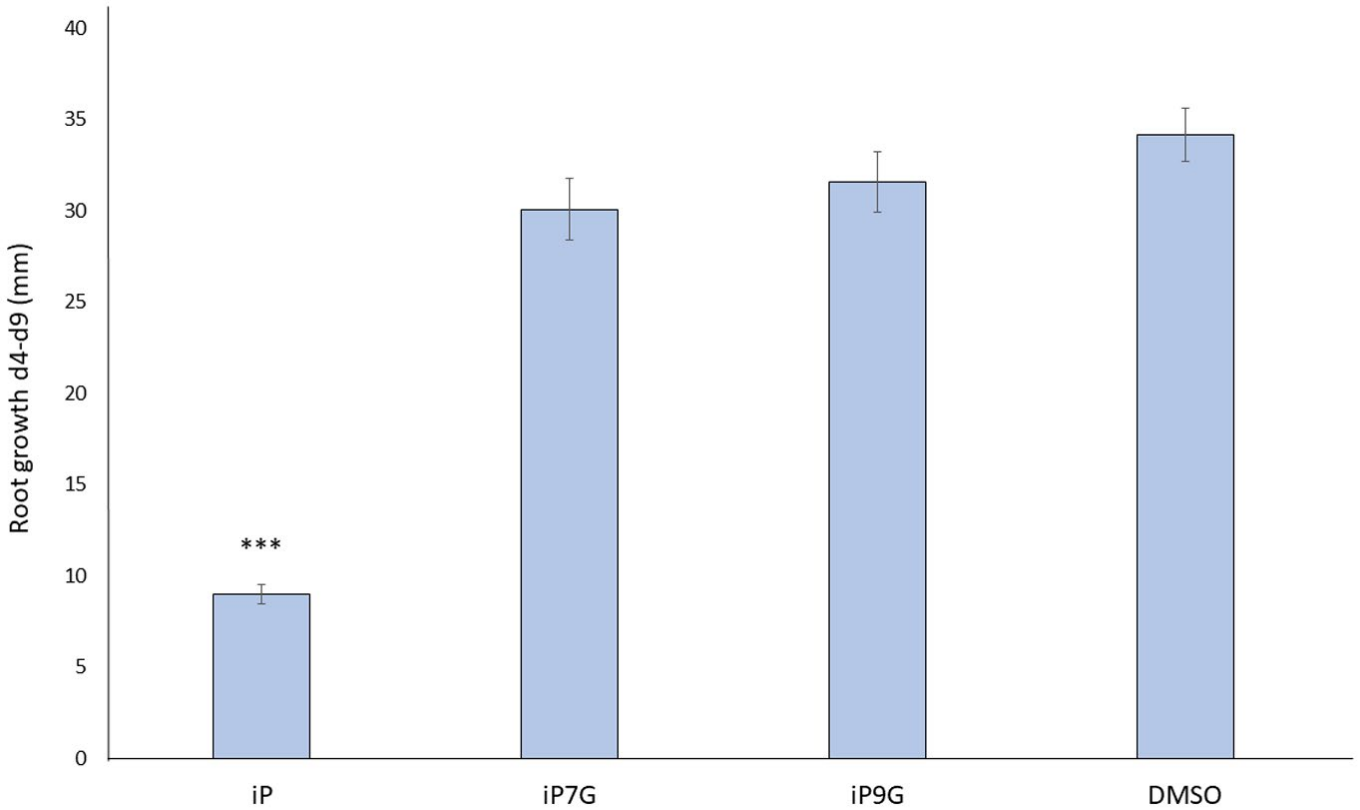
Isopentenyladenine‐N‐glucosides do not inhibit root growth. Plants were germinated vertically on standard MS + 1% sucrose and transferred to the same media supplemented with 1 µM of the indicated CK or 0.1% DMSO as a negative control on day 4. Root growth from day 4 to day 9 was measured. Data presented are averages ± SE of 4 independent biological replicates, with at least 8 plants per replicate. Student's *t*‐test, ***p < .001

To evaluate the effect of iPNGs on root growth, 4dag plate‐grown seedlings of uniform size were transferred to media containing 1 µM iP, iP7G, or iP9G, or a 0.1% DMSO control for 5 days, where root growth as length from day 4 to day 9 was measured. The high cytokinin concentration of 1 µM was chosen as it is a common concentration for these bioassays because it is a “saturating” concentration without being immediately toxic to the plants. Only plants on iP‐containing plates showed a significant reduction of root growth, approximately 74% compared to the DMSO control (Figure 3). While iP7G and iP9G treatments led to some modest reduction in root growth, 12% and 7%, respectively, neither treatment was statistically significant compared to the control (Figure 3). These data suggest iPNGs do not appear to have roles in shoot initiation nor root growth inhibition at the concentrations tested.

### 3.3 Isopentenyladenine‐9‐glucoside minorly alters gene expression in an overlapping manner as isopentenyladenine while isopenteneyl‐7‐glucoside does not

**FIGURE 4:**
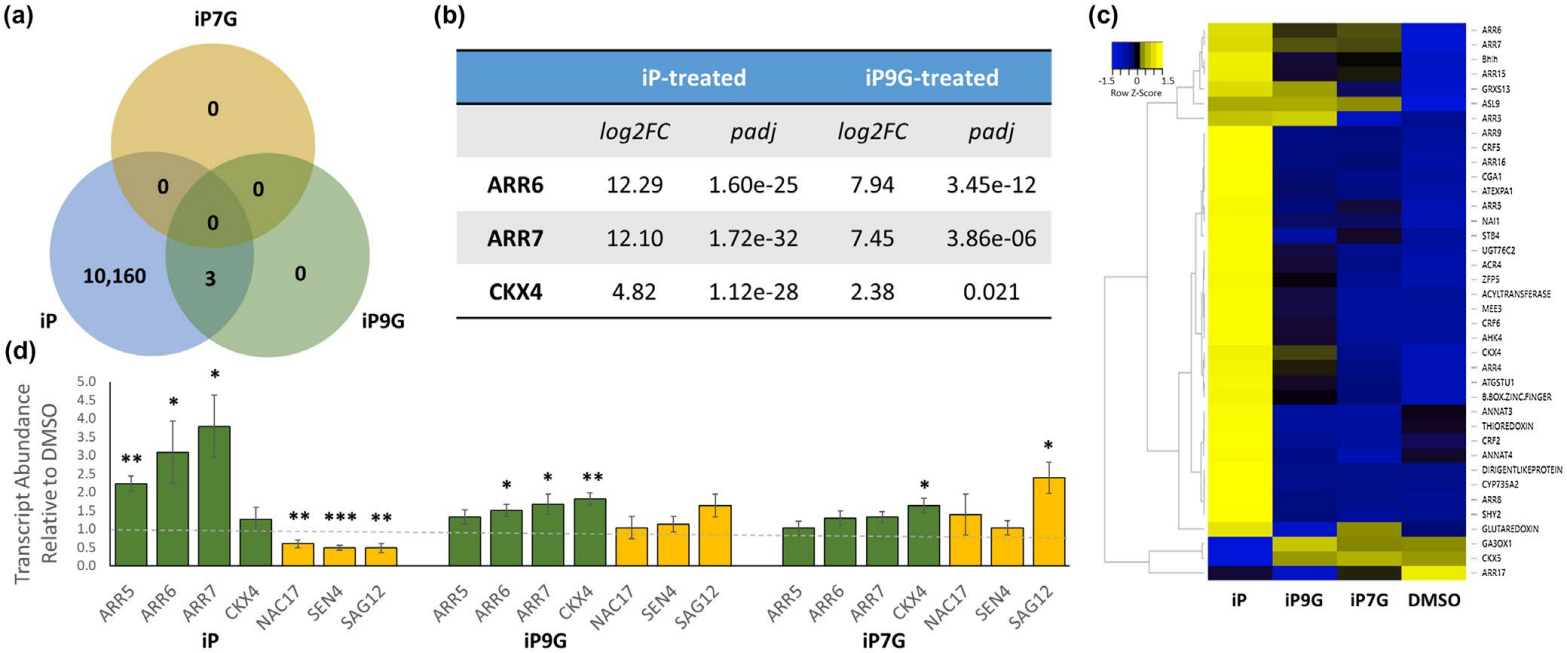
iP9G has minimal effects on the transcriptome of senescing cotyledons. Cotyledons from 12dag seedlings were floated on 3 mM MES buffer supplemented with 1 µM of indicated hormone or 0.1% DMSO as a solvent control for six days, after which RNA was extracted and sequenced. (a) Venn diagram showing the number of significantly differentially expressed genes between treatment groups as compared to the DMSO control. (b) The three DEGs altered by both iP and iP9G with log2‐based fold change and adjusted p‐value. (c) heatmap showing expression of canonically CK‐upregulated genes. (d) qPCR confirmation of RNAseq results. Green bars represent genes typically upregulated by CK treatment, yellow bars represent genes upregulated during leaf senescence. Student's *t*‐test was performed between CK and DMSO for each gene and significance noted above each bar, *p < .05 **p < .01 ***p < .001

To determine if iPNGs regulated gene expression similarly to iP during cotyledon senescence bioassays, tissue was collected at the end of cotyledon senescence bioassays and snap frozen. RNA was extracted and RNA‐sequencing (RNAseq) was performed on cotyledons after 6d dark treatment with 1 µM iP, iP7G, iP9G, and 0.1% DMSO. Three replicates of each treatment were performed, and at least twenty million paired‐end reads per replicate were generated; reads were mapped back to the Arabidopsis reference genome with a mapping rate >97% for every sample (Anders et al., 2015; Cheng et al., 2017; Pertea et al., 2016). Differential Expression Analysis was performed using DESeq2 (Love et al., 2014). More than 10,000 differentially expressed genes (DEGs) were identified between iP and DMSO (Figure 4a). Surprisingly, only three DEGs were identified between iP9G and DMSO, and no DEGs between iP7G versus DMSO (Figure 4a). Although there were a large number of genes that showed differences in fold change after treatment with iP9G and iP7G, there were no statistically significant DEGs for iP7G and only three for iP9G. Interestingly, the three statistically significant iP9G DEGs were each key CK response genes: type‐A response regulators ARR6, ARR7, and CK degrading enzyme CKX4. Each of these genes appears on the “Golden List” of transcripts routinely upregulated by CK treatment (Bhargava et al., 2013). Based on a previous meta‐analysis, a list of 38 genes was identified which show nearly universal upregulation following CK treatment (Bhargava et al., 2013). This subset of “Golden List” genes were upregulated by CK treatment in at least six microarray experiments and were also validated by a method other than microarray such as RNA‐seq or qRT‐PCR (Bhargava et al., 2013). While nearly all of these 38 genes are significantly upregulated by iP treatment in this analysis, only a few show upregulation by iP9G or iP7G compared to DMSO (Figure 4c). Most of these changes are not as dramatic as what is seen with iP treatment and as noted only three were found as having significant *p*adj levels for iP9G (Figure 4). Additionally, qRT‐PCR confirmation of RNAseq results was performed on common CK‐responsive genes, especially the three iP9G regulated gene as well as markers of leaf senescence including SAG12, SEN4, and NAC17 (Figure 4d). As expected, CK‐responsive genes were upregulated in iP while senescence genes were significantly downregulated (Figure 4d). Though the effects were mild, iP9G DEGs were are also found to be significantly upregulated for ARR6, ARR7, and CKX4 by qPCR (Figure 4d). Interestingly, another CK‐responsive gene ARR5 is significantly upregulated by iP, but not iP9G or iP7G.

**FIGURE 5:**
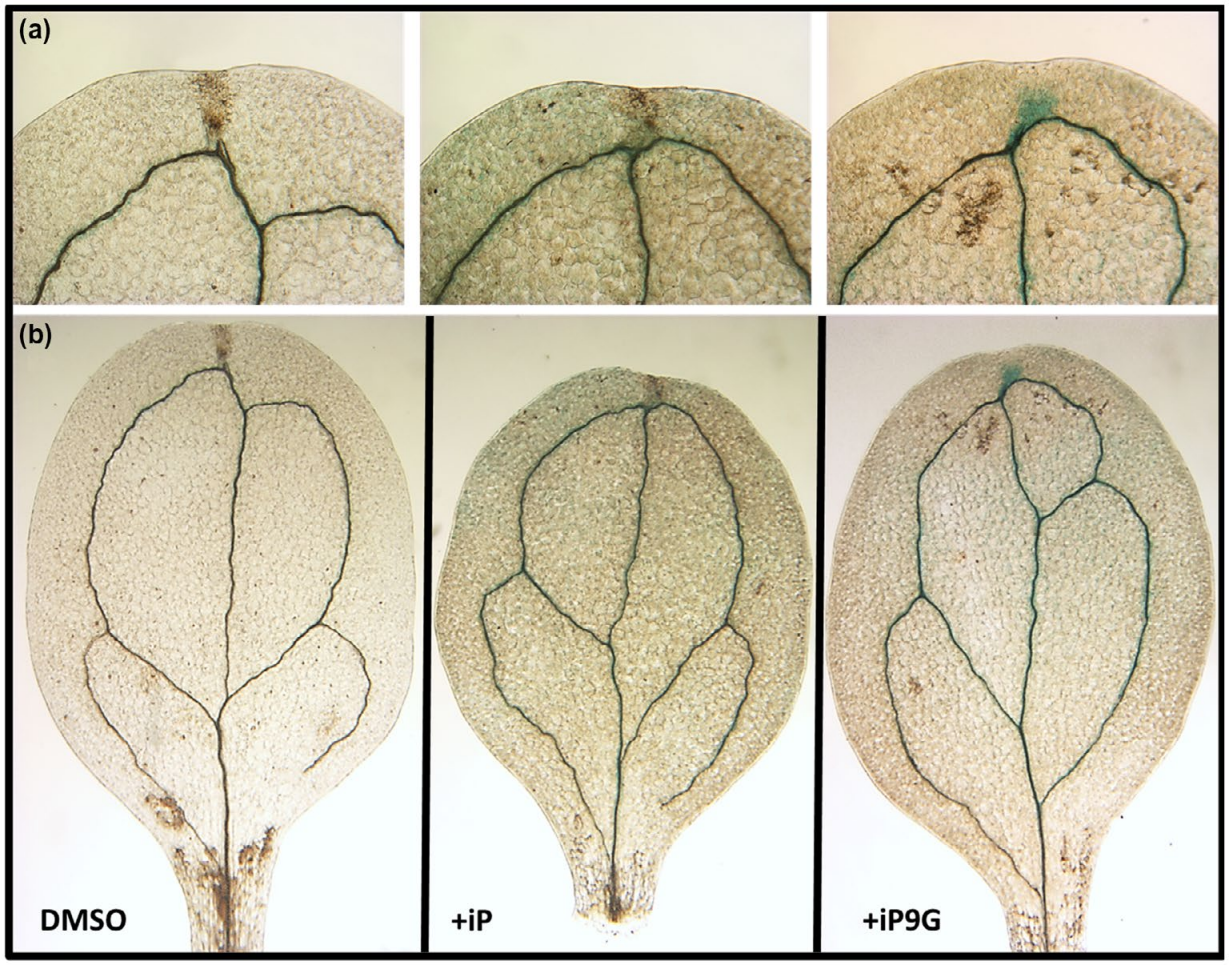
ARR6 is induced by iP and iP9G. Induction of promoterARR6::GUS is seen primarily around vascular tissue in detached cotyledons after the senescence assay. Cotyledons from 12dag seedlings were floated on 3 mM MES buffer supplemented with 1.0 µM of iP or iP9G hormone or 0.1% DMSO as a solvent control then GUS stained and cleared. (a) a magnified view of the same cotyledon in (b) Representative images are shown from at least 10 cotyledons from three independent replicates

To further investigate the link between iP and iP9G upregulation of ARR6 during senescence found from these transcript results, the promoter ARR6 reporter line pARR6::GUS was examined (Figure 5). pARR6::GUS plants were used in a cotyledon senescence assay with iP, iP9G, or DMSO as done for chlorophyll, RNAseq and qPCR analyses then stained and examined for GUS expression. While ARR6 staining levels were quite low after senescence treatment with DMSO, they are visible around vascular tissue upon close inspection (Figure 5a). Both iP and iP9G showed increased levels of ARR6 GUS staining primarily around vascular tissues over that seen with DMSO, which is consistent with the transcript upregulation. Interestingly, iP9G treatment showed additional ARR6 GUS staining at the tip of the cotyledon that was not seen in either iP or DMSO treated cotyledons (Figure 5).

**FIGURE 6:**
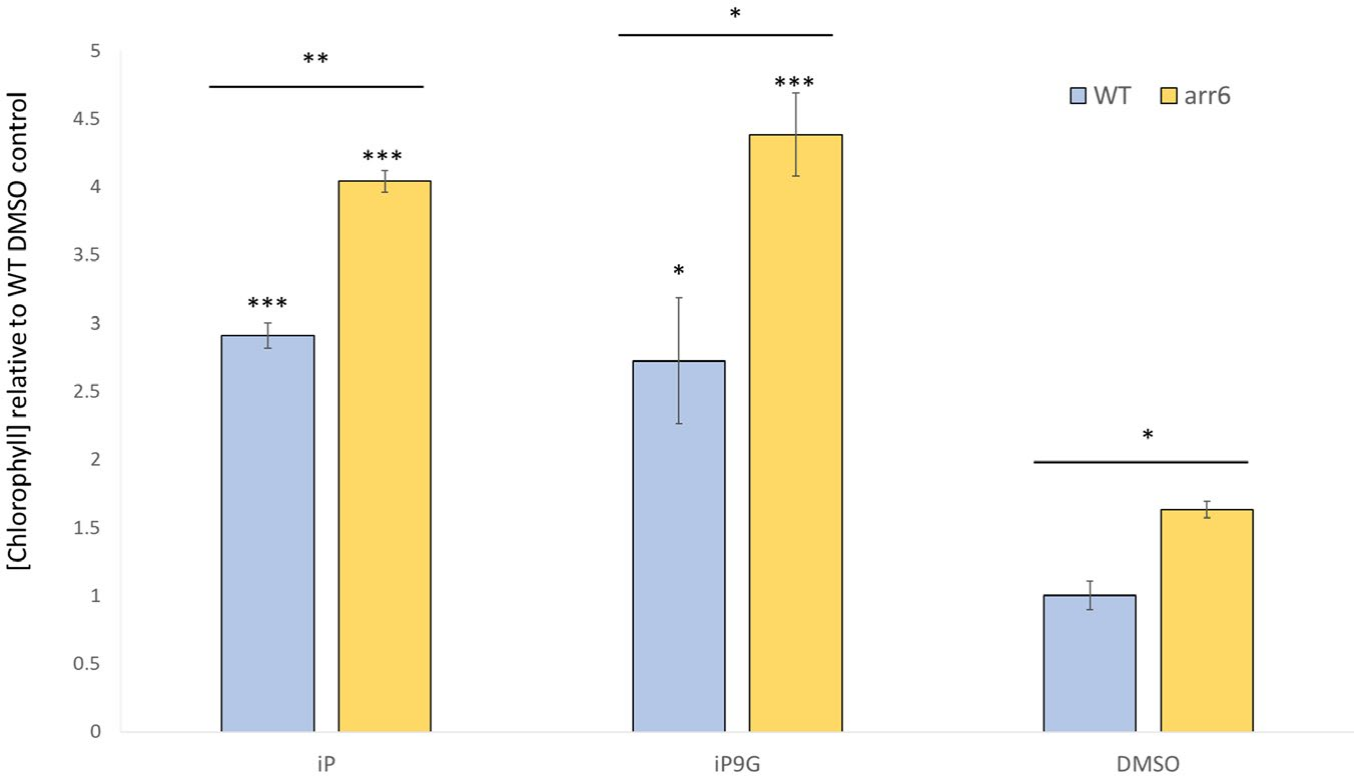
Isopentenyladenine and its 9‐glucoside delays senescence to a greater extent in detached cotyledons of *arr6* mutant. Cotyledons from 12dag seedlings of WT or *arr6* were floated on 3 mM MES buffer supplemented with 1 μM of indicated hormone or 0.1% DMSO as a solvent control. Average ± SE of three independent replicates. Student's *t*‐test was performed between CK and DMSO in each genotype noted above each bar and between *arr6* and WT of similar treatments noted with the line above each treatment, *p < .05 **p < .01 ***p < .001

One additional examination of the connection between iP, iP9G, and ARR6 during senescence was conducted by examining changes in chlorophyll levels of cotyledon senescence in this same cotyledon senescence assay with a knockout ARR6 mutant (*arr6*) versus WT (Figure 6). As seen for WT cotyledons in Figure 1, both iP and iP9G significant delay senescence and have levels of chlorophyll 2–3 times higher than the DMSO treatment control. If ARR6 had no effect on this process we would predict that the *arr6* mutant would have a similar response to WT in this assay. Instead, chlorophyll levels were higher in *arr6* with DMSO, and iP and iP9G strongly upregulate levels about 50% higher than in WT (Figure 6). Together with the GUS staining results these findings suggest a link between ARR6 and cytokinin‐regulated senescence response with iP and iP9G.

### 3.4 Isopentenyladenine treatment significantly alters expression of senescence‐associated transcription factors

Leaf senescence is a tightly regulated process which relies on highly coordinated gene regulation (Lim et al., 2007). As such, proper expression of TFs, and as a result their target genes, is necessary for senescence to occur. To determine if any previously identified senescence‐responsive or senescence‐mediating TFs were altered by iP treatment, a list of 48 TFs was generated and the effect of iP was evaluated (Chen et al., 2002; Kim et al., 2013, 2018; Miao et al., 2004; Ülker et al., 2007; Yang et al., 2011; Zwack et al., 2013; Table 1). All TFs in the list have shown upregulation during leaf senescence, with the exception of CRF6 whose expression decreases during senescence (Zwack et al., 2013). Just over half (25/48) of the TFs were significantly regulated by iP treatment as compared to the DMSO control, and a majority of those (21/25) displayed expression patterns opposite to what is typically seen during senescence (Table 1).

**TABLE 1:** Treatment with iP leads to downregulation of many TFs typically upregulated during senescence

AGI	Gene Name	log2FC iP	log2FC iP7G	log2FC iP9G	Previous identifying study
AT3G28210	SAP12	**−4.31**	−0.18	−0.09	Chen et al. (2002)
AT2G28710	AT2G28710	**−3.15**	−0.08	−0.24	Chen et al. (2002)
AT2G46680	HB−7	**−3.07**	0.03	−0.15	Chen et al. (2002)
AT2G47190	MYB2	**−3.06**	0.08	−0.14	Chen et al. (2002)
AT1G62300	WRKY6	**−2.74**	−0.09	−0.16	Chen et al. (2002)
AT1G56650	PAP1	−2.41	−0.42	0.36	Chen et al. (2002)
AT3G23250	ATMYB15	**−2.21**	0.15	0.06	Chen et al. (2002)
AT1G06180	MYB13	**−2.10**	−0.13	0.00	Chen et al. (2002)
AT4G39780	AT4G39780	**−2.05**	0.14	−0.04	Chen et al. (2002)
AT5G47230	ERF5	**−1.64**	0.06	−0.02	Chen et al. (2002)
AT1G46768	RAP2.1	**−1.53**	−0.10	−0.24	Chen et al. (2002)
AT3G51960	BZIP24	**−1.25**	−0.06	−0.33	Chen et al. (2002)
AT2G27580	SAP3	**−1.22**	0.01	−0.16	Chen et al. (2002)
AT5G65210	TGA1	**−1.19**	−0.04	−0.14	Chen et al. (2002)
AT1G30490	PHV	**−1.17**	−0.02	−0.05	Chen et al. (2002)
AT5G06960	OBF5	**−0.79**	−0.08	−0.08	Chen et al. (2002)
AT3G14230	RAP2.2	**−0.79**	−0.02	−0.06	Chen et al. (2002)
AT4G34990	MYB32	−0.75	0.05	−0.01	Chen et al. (2002)
AT5G06100	MYB33	**−0.71**	0.01	−0.06	Chen et al. (2002)
AT2G40950	BZIP17	**−0.67**	0.02	0.11	Chen et al. (2002)
AT4G01910	AT4G01910	**−0.65**	0.03	−0.05	Chen et al. (2002)
AT1G34190	NAC017	**−0.58**	0.05	−0.01	Kim et al. (2018)
AT3G01470	HB−1	−0.46	−0.03	−0.07	Chen et al. (2002)
AT4G11680	AT4G11680	−0.41	0.11	0.07	Chen et al. (2002)
AT5G10030	TGA4	−0.36	−0.04	−0.17	Chen et al. (2002)
AT2G23340	DEAR3	−0.35	0.08	0.09	Chen et al. (2002)
AT2G25000	WRKY60	−0.35	−0.03	0.15	Chen et al. (2002)
AT1G13960	WRKY4	−0.34	−0.04	0.05	Chen et al. (2002)
AT5G13180	VNI2	−0.22	0.03	0.04	Yang et al. (2011)
AT1G78080	RAP2.4	0.15	0.20	0.24	Chen et al. (2002)
AT4G01250	WRKY22	0.16	−0.01	0.04	Zhou et al. (2011)
AT2G46270	GBF3	0.19	−0.16	0.00	Chen et al. (2002)
AT3G23240	ERF1	0.22	0.06	0.18	Chen et al. (2002)
AT1G13260	RAV1	0.26	−0.19	−0.05	Woo et al. (2016)
AT5G09330	NAC082	0.30	0.01	0.01	Kim et al. (2018)
AT4G23810	WRKY53	0.38	−0.10	0.26	Miao et al. (2004)
AT4G24240	WRKY7	0.40	−0.30	−0.03	Chen et al. (2002)
AT3G56400	WRKY70	0.49	−0.06	−0.10	Ülker et al. (2007)
AT2G31180	MYB14	0.57	0.56	0.02	Chen et al. (2002)
AT5G22380	NAC090	0.57	0.77	1.91	Kim et al. (2018)
AT1G34180	NAC016	**0.61**	0.02	0.14	Kim et al. (2013)
AT4G01720	WRKY47	0.64	−0.18	0.18	Chen et al. (2002)
AT2G33310	IAA13	**0.72**	0.02	−0.01	Chen et al. (2002)
AT2G02820	MYB88	**0.82**	0.02	−0.16	Chen et al. (2002)
AT4G38620	MYB4	1.19	−0.04	−0.08	Chen et al. (2002)
AT3G46130	MYB48	1.61	−0.81	0.50	Chen et al. (2002)
AT3G61630	CRF6	**2.23**	0.03	0.56	Zwack et al. (2013) (CRF6 is downregulated during senescence)
AT2G01940	SGR5	**2.31**	0.23	−0.16	Chen et al., (2002)

Note

All TFs listed in this table, except CRF6, have been previously reported to be upregulated during leaf senescence and/or play roles in leaf senescence. Approximately half of the list show downregulation as noted by blue colored cells. Darker blue and darker yellow indicate more severe down‐ and upregulation, respectively. The log2FC is log‐2‐based fold change relative to DMSO control. Bold‐faced values indicate the differential expression is statistically significant (*p*adj < .05).

Of particular interest are CRF6, NAC017, and WRKY6. CRF6 is a CK‐responsive TF which negatively regulates senescence and is downregulated during senescence (Zwack et al., 2012, 2013). Interestingly, CRF6 is also induced by oxidative stress and promotes stress tolerance by modulating the expression of CK‐associated genes (Zwack et al., 2016). Treatment with iP led to significant upregulation of CRF6 (Table 1). Notably, CRF6 is a direct target of NAC017 (Ng et al., 2013). NAC017 is one member of several NAC TFs involved in senescence response and is known to be upregulated during leaf senescence (Kim et al., 2018). Further study of NAC017 has identified it as a key player in organellar stress response and retrograde signaling, and it is an activator of many genes involved in senescence, cell death, and autophagy (Meng et al., 2019). WRKY6 was identified in a screen for transcriptionally upregulated TFs during leaf senescence, and deeper analysis has characterized it as being a positive regulator of leaf senescence by directly activating expression of senescence genes; overexpression of WRKY6 leads to an early senescent phenotype (Chen et al., 2002; Robatzek & Somssich, 2002; Zhang et al., 2018). Both NAC017 and WRKY6 are significantly downregulated by iP treatment (Table 1).

### 3.5 Isopentenyladenine upregulates genes involved in photosynthesis and ribosome biogenesis while downregulating senescence and catabolism associated genes

**FIGURE 7:**
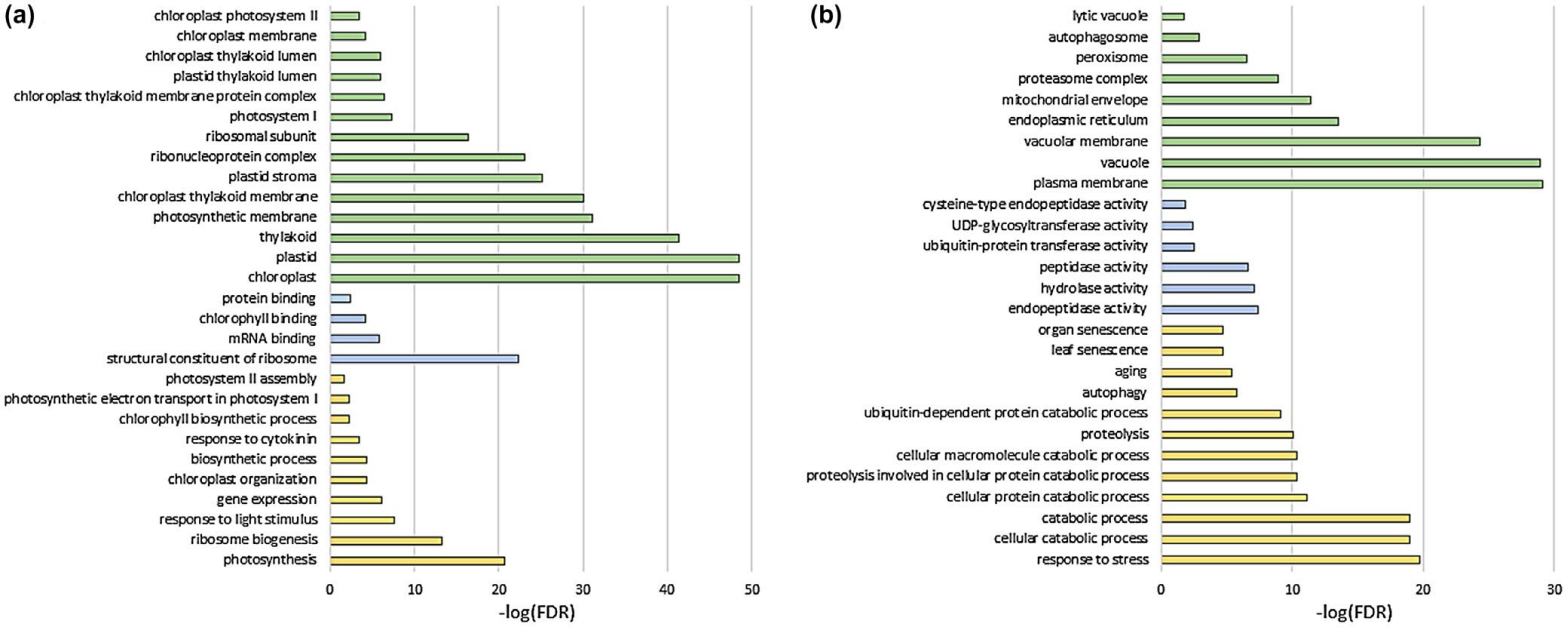
iP treatment of senescing cotyledons has significant effects on transcriptional regulation. Select GO terms significantly enriched in (a) iP‐upregulated and (b) iP‐downregulated list. Green bars associated with Cell Part GO terms, Blue with Molecular Function GO terms, Gold with Biological Process GO terms

After identifying key senescence TFs possibly responsible for the high number of DEGs seen between iP and DMSO treatment, further analysis of the 10,163 DEGs was performed to identify potential mechanisms by which iP may be delaying senescence in detached cotyledons. Gene Ontology (GO) enrichment analysis was performed on both upregulated and downregulated gene lists. GO terms significantly enriched in the upregulated gene lists include cell components such as “chloroplast,” “ribosomal subunit,” and “chloroplast photosystem II”; enriched molecular functions include “structural constituent of ribosome” and “chlorophyll binding”; and enriched biological processes include “photosynthesis,” “ribosome biogenesis,” “response to cytokinin,” and “photosystem II assembly” (Figure 7a). GO terms significantly enriched in the downregulated gene lists include cell parts such as “authophagosome” and “proteasome complex”; enriched molecular functions include “endopeptidase activity,” “hydrolase activity,” and “ubiquitin‐protein transferase activity”; enriched biological processes include “proteolysis,” “catabolic process,” and “leaf senescence” (Figure 7b). These data indicate significant roles for iP in delaying leaf senescence by upregulating photosynthetic and translation machinery during CK response, while downregulating catabolic processes like proteolysis and autophagy often associated with senescence and cell death (Bassham, 2007; Hildebrandt et al., 2015).

To evaluate the physiological impacts of changes seen from transcriptomic analysis of cotyledons from 6d dark senescence assays, three examinations were performed. First, cotyledons from this assay were measured for chlorophyll fluorescence as a proxy for photosynthetic response and photosystem II integrity, as genes associated with photosynthesis were significantly upregulated by iP treatment. As expected, cotyledons treated with iP have significantly higher photosystem II efficiency as determined by Fv/Fm compared to that of DMSO control (Figure S2a). Second, iP‐treated cotyledons were examined for overall protein levels, as genes associated with translation and ribosome biogenesis were significantly upregulated by iP treatment. The iP‐treated cotyledons were found to have higher protein content than their DMSO counterparts (Figure S2b). Finally, amounts of cell death was examined in post‐senescence assay cotyledons with Trypan Blue vital staining, as genes associated with senescence and cell death were significantly downregulated by iP treatment. iP‐treated cotyledons appear to have the least staining of any treatment while DMSO‐treated cotyledons stained the most heavily, with both iP7G and iP9G appear to have an intermediate amount of staining (Figure S2c).

## 4 DISCUSSION

### 4.1 Isopentenyladenine‐N‐glucosides do not mimic isopentenyladenine in most bioassays

Active CKs have canonical activities such as inhibiting root growth, promoting shoot regeneration, and delaying senescence (Kieber & Schaller, 2014; Mok & Mok, 2001; Skoog & Miller, 1957). In each of these CK‐bioassays conducted in this study, the CK base iP has strong and standard CK functional activity, similar to other CK examined in similar assays (Figures 1, 2, 3). In direct contrast, the iP N‐glucoside iP7G did not have significant effects in any of these bioassays examined here at the tested concentration (Figures 1, 2, 3). While this cannot fully rule out potential CK activity or functions for iP7G in any of these processes, as higher concentrations of iP7G could be further examined. The concentrations used in this study were chosen because they are on the higher end of what is commonly used in CK assays, but considering the fact that iP7G is routinely measured at orders of magnitude more highly concentrated than iP (Hošek et al., 2020; Kiba et al., 2019; Nishiyama et al., 2011; Sakakibara et al., 2005; Šimura et al., 2018; Svačinová et al., 2012; Tokunaga et al., 2012), analysis of higher concentrations of exogenously applied iP7G might be more fruitful in identifying potential functions. It is worth noting that trans‐Zeatin‐N‐glucosides both showed high activity at both 1 and 0.1 µM in a detached leaf senescence assay, and these compounds have comparable endogenous concentration relative to iPNGs (Hallmark et al., 2020), potentially indicating iP7G truly does not have significant anti‐senescent capability. It is of course possible that the compounds used might not be completely pure in composition, which could also be revealed with bioassay examination at higher concentration as part of future studies. The delay of senescence by iP using this assay with a *cyp735A1,2* double mutant background that blocks conversion of iP to tZ or DHZ forms (Kiba et al., 2013), indicates that iP forms alone appear sufficient to delay senescence (Figure S1). With regards to iP9G, there was a significant 2–3 fold increase in chlorophyll retention during the detached leaf senescence assay similar to that of iP (Figure 1), but no significant activity was detected in root growth inhibition or shoot initiation (Figures 2 and 3). This may point to a role for iP9G in delaying leaf senescence, and it is noteworthy that though iP9G is often measured at much higher concentrations than iP, iP9G levels are typically significantly less than iP7G levels (Hošek et al., 2020; Kiba et al., 2013, 2019; Ko et al., 2014; Nishiyama et al., 2011; Šimura et al., 2018; Tokunaga et al., 2012). These data suggest iP9G is an active CK, albeit not as robustly active as its respective base; iP7G, however, does not appear to be an active CK as determined by these bioassays.

### 4.2 Isopentenyladenine‐9‐glucoside has minor effects on the senescing leaf transcriptome

Following a 6d detached leaf senescence experiment, iP9G treatment surprisingly only led to the significant differential expression of three genes (Figure 4a). Perhaps more surprisingly; however, is that these three genes are ARR6, ARR7, and CKX4, all of which are commonly upregulated by active CK treatments and play roles in CK signaling and degradation (Figure 4b; Bhargava et al., 2013; Galuszka et al., 2007; Rashotte et al., 2003). While these were the only three genes to be found significantly differentially expressed, several other key CK‐responsive genes showed upregulation following iP9G treatment as compared to mock treatment, including ARR4, ARR3, ARR15, CRF6, ASL9, and GRXS13 (Figure 4c). The modest upregulation of these genes by iP9G as compared with their significant upregulation by iP furthers the notion that iP9G has iP‐like activity, but this activity is decreased relative to the base.

In order to further examine potentially connections between iP9G and one of the genes found to be induced by its treatment in senescence assays transcript analysis, ARR6 was more specifically studied. First the promoter ARR6 GUS reporter line (pARR6::GUS) was examined after treatment in this cotyledon senescence assay. The pARR6::GUS line has been previously shown to be induced by CK treatment, although expression is lower in cotyledons and leaves (To et al., 2004). Here we found that pARR6::GUS levels were quite low in control DMSO treated cotyledons after this assay treatment of 6 days in the dark, although expression can be seen surrounding vascular tissue as previous seen (Figure 5; To et al., 2004). Importantly, pARR6::GUS expression can be seen as induced with both iP and iP9G primarily surrounding the vascular tissue in support of transcriptome findings (Figures 4 and 5). iP induced expression is primarily increased around vascular tissue, although there is also quite weak expression throughout the cotyledon (Figure 5). iP9G shows a similar induction around vascular tissue, but can additionally be seen at the tip of the cotyledon that is not seen with iP (Figure 5a). The broad, but quite weak expression induction of iP may explain the difference between intensity of transcript findings to iP9G, since RNA was extracted from the whole cotyledon, and not just vascular related tissue. Future investigations are needed to further resolve this, but this does establish another clear link to iP9G induction of ARR6.

A second examination of connections between ARR6, iP and iP9G and senescence were also examined by determining chlorophyll levels in an ARR6 knockout mutant background (*arr6*). Chlorophyll levels were about 50% higher in *arr6* compared to WT in the control and CK treatments (Figure 6). As ARR6 is a type‐A response regulator and functions as a negative regulator to cytokinin signaling, this base finding is consistent with previous results for this gene (To et al., 2004). The additional finding that both iP and iP9G also have higher chlorophyll levels, or more greatly delay senescence also link these CK to our findings from RNAseq and qPCR transcript results (Figures 4, 5, 6). ARR6 has been previously connected to senescence response, so these findings support previous studies (Kim et al., 2006; Li et al., 2012; To et al., 2004), but also appear to connect iP9G for the first time to this regulation, which will need additional study.

### 4.3 Isopentenyladenine treatment significantly alters the transcriptome of senescing leaves

It has long been known that active CKs delay senescence, and key regulators of CK‐delayed senescence have been previously identified. Phosphorylation of ARR2 as a result of CK binding to AHK3 has been shown to be necessary for CK to delay senescence (Kim et al., 2006). Interestingly many of these studies have used an ARR6 reporter line as the output for their findings (Kim et al., 2006). Additionally, the CK‐responsive TF CRF6 is required for CK treatment to delay senescence (Zwack et al., 2013, 2016). There has been extensive characterization of the senescing leaf transcriptome in Arabidopsis (Breeze et al., 2011; Guo et al., 2004; Kim et al., 2018; Woo et al., 2016), yet, to the authors knowledge, no transcriptome of detached leaves treated with natural CKs has been performed. A previous CK senescence transcriptome used the synthetic CK benzyladenine riboside and its halogenated forms over a 48h treatment (Vylíčilová et al., 2016).

Many TFs typically upregulated during senescence showed significant downregulation with iP treatment as compared to the DMSO control (Table 1). Several of these transcription factors including WRKY6 and NAC017 are known to positively regulate genes involved in senescence associated processes such as autophagy and cell death (Kim et al., 2018; Meng et al., 2019; Robatzek & Somssich, 2002; Zhang et al., 2018). Therefore, the repression of these TFs by iP treatment is likely one of the mechanisms by which iP treatment led to downregulation of senescence‐associated genes and inhibited leaf senescence.

Because such a large number of genes (10,163) were affected by iP treatment, gene ontology (GO) analysis was performed to gain a big‐picture view of what types of genes were affected. Because a similar number of genes were up‐ and downregulated by iP relative to DMSO (5,106 and 5,057, respectively), GO analysis was performed on both lists independently of one another (Figure 7a,b).

It has previously been noted that chloroplast and photosystem genes plays a key role in leaf senescence (Woo et al., 2016), so it is unsurprising that there is enrichment for chloroplasts and photosystems in the Cell Component GO terms (Figure 7a). Enriched Biological Process GO terms reveal some intriguing trends: upregulation of genes involved in photosynthesis, ribosome biogenesis, chlorophyll biosynthesis, response to CK, and photosystem II assembly (Figure 7a).

GO analysis of the downregulated gene list led to several interesting enriched GO terms that were distinct from the upregulated list, suggesting that CK may both induce and repress a number of processes to delay senescence. GO terms for both the Cellular Component “autophagasome” and the Biological Process “autophagy” are significantly enriched in the downregulated gene lists (Figure 7b). Autophagy is known to play an important role in leaf senescence as it is a catabolic process which aids in recycling nutrients and macromolecules (Bassham, 2007; Xiong et al., 2005). Degradation of macromolecules including proteins by endopeptidases and the proteasome are common hallmarks of senescence (Hildebrandt et al., 2015; Lim et al., 2007; Lohman et al., 1994), so unsurprisingly GO terms for the Biological Processes “catabolic process” and “proteolysis,” the Molecular Functions “endopeptidase activity” and “ubiquitin‐protein transferase activity,” and the Cellular Component “proteasome complex” and “lytic vacuole” are enriched in the downregulated gene list. Important to note is the GO term enrichment for “aging” and “leaf senescence” in the downregulated gene list as one would expect. Catabolism of proteins and ultimately amino acids is a consequence of senescence as cells use the breakdown of these molecules for energy (Avin‐Wittenberg et al., 2015; Hildebrandt et al., 2015).

### 4.4 Examination of cotyledons after senescence bioassays supports transcriptome findings

As many biological processes were affected by iP treatment of iP as a CK according to transcriptome findings, we decided to assay some of these processes *in planta* following the detached leaf senescence assays. One of the most significantly upregulated biological processes in this assay by iP was photosynthesis, specifically photosystem II assembly (Figure 7a). It had already been determined that iP treatment led to higher chlorophyll retention than DMSO treatment in these assays (Figure 1), however, measurement of chlorophyll fluorescence can determine the efficiency of photosystem II. Based on transcriptome results, one would expect higher photosystem II efficiency in iP treatment relative to DMSO, and this result was borne out in Fv/Fm measurements revealing iP‐treated cotyledons had approximately double the Fv/Fm of that of DMSO‐treated cotyledons (Figure S2a). However, this was not borne out for iP9G, where there was a lot of variability from the parallel examination, suggesting further work on this is needed.

Genes involved in ribosome biogenesis were found to be significantly upregulated while genes involved in various protein degradation processes including proteasome‐mediated degradation and autophagy were downregulated. Attempts to determine if gene expression and protein levels were similar, suggest potentially similar regulation by iP and iPNGs, although addition efforts are needed to fully discern this.

Treatment of cotyledons with iP led to a significant downregulation of genes associated with leaf senescence and cell death. To determine if these transcriptional changes led to a change in cell death, Trypan blue staining was performed and revealed limited cell death as compared to the DMSO control (Figure S2c). Treatment with iPNGs appeared to also lead to less cell death than DMSO, though to a lesser degree than iP. Again, these findings support both the transcriptome findings (Figure 4) and support the role for iP in delaying senescence and cell death in detached cotyledons.

### 4.5 iPNGs are not identical in their activity to tZ‐N‐glucosides

Previous work with tZ‐N‐glucosides (tZNGs) revealed the compounds are capable of delaying senescence in senescence assays similarly to iP9G, but, like iPNGs, tZNGs had minimal to no effect on root growth or shoot regeneration (Hallmark et al., 2020). While the conditions of the transcriptome experiment differ greatly, it appears that there may be stark differences between tZNGs and iPNGs in the number of DEGs, with a two‐hour treatment with tZNGs led to hundreds of DEGs (Hallmark et al., 2020), while a multi‐day senescence assay revealed only three DEGs were identified with iP9G treatment and zero with iP7G (Figure 4a). Regardless of transcriptome differences, the ability of iP9G and the tZNGs to delay senescence in detached leaves may indicate a role for CKNGs collectively in delaying senescence or regulating levels of CK bases.

### 4.6 Mechanism of iP9G activity remains unclear

While showing some activity in the detached leaf chlorophyll retention assay (Figure 1) along with upregulation of CK‐related genes in the senescence transcriptome (Figure 4), the exact mechanism by which iP9G displays activity is unclear. As previously reported, conversion of CK‐N‐glucosides to CK bases is unlikely (Hallmark et al., 2020; Hošek et al., 2020; Palni et al., 1984). As iP9G appears to act as a less active form of iP at the transcriptional level (Figure 4), it appears that iP9G is activating the cytokinin response pathway, at least during the senescence assay. We believe that there are two non‐mutually exclusive possible mechanisms to explain this. First, exogenous treatment with iP9G may make it unfavorable for iP base to be converted to iP9G, leading to an increase in iP levels and concomitantly activation of the cytokinin signaling pathway. Second, iP9G may directly bind CK receptors albeit with much lower affinity than iP. Previous tests of Arabidopsis CK receptor binding activity have not included iP‐N‐glucosides (Romanov et al., 2006; Spíchal et al., 2004; Yamada et al., 2001). There is also the issue of subcellular localization. CKNGs have been previously shown to be located largely in the apoplast and vacuole in leaves (Jiskrová et al., 2016), while CK receptors are primarily localized to the endoplasmic reticulum (Caesar et al., 2011; Wulfetange et al., 2011). Recent work, though, suggests CK receptors also appear to be present at the plasma membrane under certain conditions (Kubiasová et al., 2020), possibly placing them in a better position to interact with CKNGs.

Whatever the mechanism, it is clear iP9G has CK activity during senescence which has largely gone previously unrecognized. While glycosylation of iP certainly dampens its effects, data presented here suggest this glycosylation is not fully inactivating, particularly at the N9 position. Moving forward, cytokinin biologists should reassess the generalization that CK‐N‐glucosides are biologically fully inert at least in relation to senescence response and consider that CK activities previously thought to be performed by CK bases alone could also be performed by their respective glucosides or in combination.

## CONFLICT OF INTEREST

The authors declare no conflict of interest.

## AUTHOR CONTRIBUTIONS

HTH designed the study, conducted the experiments, analyzed data, and wrote the manuscript. AMR secured funding, designed the study, analyzed data, and wrote the manuscript.

## Supporting information

Figure S1

Figure S2

Table S1

## References

[pld3292-bib-0001] Anders, S. , Pyl, P. T. , & Huber, W. (2015). HTSeq—a Python framework to work with high‐throughput sequencing data. Bioinformatics, 31, 166–169. 10.1093/bioinformatics/btu638 25260700 PMC4287950

[pld3292-bib-0002] Andrews, S. (2010). FastQC: A quality control tool for high throughput sequence data. http://www.bioinformatics.babraham.ac.uk/projects/fastqc/

[pld3292-bib-0003] Avin‐Wittenberg, T. , Bajdzienko, K. , Wittenberg, G. , Alseekh, S. , Tohge, T. , Bock, R. , Giavalisco, P. , & Fernie, A. R. (2015). Global analysis of the role of autophagy in cellular metabolism and energy homeostasis in arabidopsis seedlings under carbon starvation. The Plant Cell, 27, 306–322. 10.1105/tpc.114.134205 25649436 PMC4456922

[pld3292-bib-0004] Babicki, S. , Arndt, D. , Marcu, A. , Liang, Y. , Grant, J. R. , Maciejewski, A. , & Wishart, D. S. (2016). Heatmapper: Web‐enabled heat mapping for all. Nucleic Acids Research, 44, W147–W153. 10.1093/nar/gkw419 27190236 PMC4987948

[pld3292-bib-0005] Bassham, D. C. (2007). Plant autophagy—more than a starvation response. Current Opinion in Plant Biology, 10, 587–593. 10.1016/j.pbi.2007.06.006 17702643

[pld3292-bib-0006] Bhargava, A. , Clabaugh, I. , To, J. P. , Maxwell, B. B. , Chiang, Y.‐H. , Schaller, G. E. , Loraine, A. , & Kieber, J. J. (2013). Identification of cytokinin‐responsive genes using microarray meta‐analysis and RNA‐Seq in arabidopsis. Plant Physiology, 162, 272–294. 10.1104/pp.113.217026 23524861 PMC3641208

[pld3292-bib-0007] Biddington, N. L. , & Thomas, T. H. (1978). Influence of different cytokinins on the transpiration and senescence of excised oat leaves. Physiologia Plantarum, 42, 369–374. 10.1111/j.1399-3054.1978.tb04098.x

[pld3292-bib-0008] Bolger, A. M. , Lohse, M. , & Usadel, B. (2014). Trimmomatic: A flexible trimmer for Illumina sequence data. Bioinformatics, 30, 2114–2120. 10.1093/bioinformatics/btu170 24695404 PMC4103590

[pld3292-bib-0009] Breeze, E. , Harrison, E. , McHattie, S. , Hughes, L. , Hickman, R. , Hill, C. , Kiddle, S. , Kim, Y. , Penfold, C. A. , Jenkins, D. et al (2011). High‐resolution temporal profiling of transcripts during arabidopsis leaf senescence reveals a distinct chronology of processes and regulation. The Plant Cell, 23, 873–894. 21447789 10.1105/tpc.111.083345 PMC3082270

[pld3292-bib-0010] Caesar, K. , Thamm, A. M. K. , Witthöft, J. , Elgass, K. , Huppenberger, P. , Grefen, C. , Horak, J. , & Harter, K. (2011). Evidence for the localization of the Arabidopsis cytokinin receptors AHK3 and AHK4 in the endoplasmic reticulum. Journal of Experimental Botany, 62, 5571–5580. 10.1093/jxb/err238 21841169 PMC3223052

[pld3292-bib-0011] Chen, W. , Provart, N. J. , Glazebrook, J. , Katagiri, F. , Chang, H.‐S. , Eulgem, T. , Mauch, F. , Luan, S. , Zou, G. , Whitham, S. A. , Budworth, P. R. , Tao, Y. I. , Xie, Z. , Chen, X. I. , Lam, S. , Kreps, J. A. , Harper, J. F. , Si‐Ammour, A. , Mauch‐Mani, B. , … Zhu, T. (2002). Expression profile matrix of arabidopsis transcription factor genes suggests their putative functions in response to environmental stresses. The Plant Cell, 14, 559–574. 10.1105/tpc.010410 11910004 PMC150579

[pld3292-bib-0012] Cheng, C. , Krishnakumar, V. , Chan, A. , Thibaud‐Nissen, F. , Schobel, S. , & Town, C. (2017). Araport11: A complete reannotation of the Arabidopsis thaliana reference genome. The Plant Journal, 89, 789–804. 27862469 10.1111/tpj.13415

[pld3292-bib-0013] Deleuze, G. G. , McChesney, J. D. , & Fox, J. E. (1972). Identification of a stable cytokinin metabolite. Biochemical and Biophysical Research Communications, 48, 1426–1432. 10.1016/0006-291X(72)90872-8 5077828

[pld3292-bib-0014] Fernández‐Bautista, N. , Domínguez‐Núñez, J. , Moreno, M. M. , & Berrocal‐Lobo, M. (2016). Plant Tissue Trypan Blue Staining During Phytopathogen Infection. BIO‐PROTOCOL, 6, 2078 10.21769/BioProtoc.2078

[pld3292-bib-0015] Gajdošová, S. (2011). Biological effects and metabolism of cis‐Zeatin‐type cytokinins in plants. Prague, Czech Republic: Charles University.

[pld3292-bib-0016] Galuszka, P. , Popelková, H. , Werner, T. , Frébortová, J. , Pospíšilová, H. , Mik, V. , Köllmer, I. , Schmülling, T. , & Frébort, I. (2007). Biochemical characterization of cytokinin oxidases/dehydrogenases from arabidopsis thaliana expressed in *Nicotiana tabacum* L. Journal of Plant Growth Regulation, 26, 255–267. 10.1007/s00344-007-9008-5

[pld3292-bib-0017] Gan, S. , & Amasino, R. M. (1996). Cytokinins in plant senescence: From spray and pray to clone and play. BioEssays, 18, 557–565. 10.1002/bies.950180707

[pld3292-bib-0018] Glazebrook, J. , & Weigel, D. (2002). Arabidopsis: A laboratory manual. Cold Spring Harbor Laboratory Press.

[pld3292-bib-0100] Gordon, S. P. , Heisler, M. G. , Reddy, G. V. , Ohno, C. , Das, P. , & Meyerowitz, E. (2007). Pattern formation during de novo assembly of the Arabidopsis shoot meristem. Development, 134, 3539–3548. 17827180 10.1242/dev.010298

[pld3292-bib-0019] Guo, Y. , Cai, Z. , & Gan, S. (2004). Transcriptome of arabidopsis leaf senescence. Plant, Cell and Environment, 27, 521–549. 10.1111/j.1365-3040.2003.01158.x

[pld3292-bib-0020] Hallmark, H. T. , Černý, M. , Brzobohatý, B. , & Rashotte, A. M. (2020). trans‐Zeatin‐N‐glucosides have biological activity in Arabidopsis thaliana. PLoS One, 15, e0232762 10.1371/journal.pone.0232762 32379789 PMC7205299

[pld3292-bib-0021] Hildebrandt, T. M. , Nunes Nesi, A. , Araújo, W. L. , & Braun, H.‐P. (2015). Amino acid catabolism in plants. Molecular Plant, 8, 1563–1579. 10.1016/j.molp.2015.09.005 26384576

[pld3292-bib-0022] Hörtensteiner, S. (2006). Chlorophyll degradation during senescence. Annual Review of Plant Biology, 57, 55–77. 10.1146/annurev.arplant.57.032905.105212 16669755

[pld3292-bib-0023] Hošek, P. , Hoyerová, K. , Kiran, N. S. , Dobrev, P. I. , Zahajská, L. , Filepová, R. , Motyka, V. , Müller, K. , & Kamínek, M. (2020). Distinct metabolism of N‐glucosides of isopentenyladenine and trans‐zeatin determines cytokinin metabolic spectrum in Arabidopsis. New Phytologist, 225, 2423–2438. 10.1111/nph.16310 31682013

[pld3292-bib-0024] Hou, B. , Lim, E.‐K. , Higgins, G. S. , & Bowles, D. J. (2004). N‐Glucosylation of cytokinins by glycosyltransferases of arabidopsis thaliana. Journal of Biological Chemistry, 279, 47822–47832. 10.1074/jbc.M409569200 15342621

[pld3292-bib-0025] Hwang, I. , Sheen, J. , & Müller, B. (2012). Cytokinin signaling networks. Annual Review of Plant Biology, 63, 353–380. 10.1146/annurev-arplant-042811-105503 22554243

[pld3292-bib-0026] Jiskrová, E. , Novák, O. , Pospíšilová, H. , Holubová, K. , Karády, M. , Galuszka, P. , Robert, S. , & Frébort, I. (2016). Extra‐ and intracellular distribution of cytokinins in the leaves of monocots and dicots. New Biotechnology, 33, 735–742. 10.1016/j.nbt.2015.12.010 26777983

[pld3292-bib-0027] Keshishian, E. A. , & Rashotte, A. M. (2015). Plant cytokinin signalling. Essays in Biochemistry, 58, 13–27. 10.1042/bse0580013 26374884

[pld3292-bib-0028] Kiba, T. , Takebayashi, Y. , Kojima, M. , & Sakakibara, H. (2019). Sugar‐induced de novo cytokinin biosynthesis contributes to Arabidopsis growth under elevated CO 2. Scientific Reports, 9, 1–15. 10.1038/s41598-019-44185-4 31123308 PMC6533260

[pld3292-bib-0029] Kiba, T. , Takei, K. , Kojima, M. , & Sakakibara, H. (2013). Side‐chain modification of cytokinins controls shoot growth in arabidopsis. Developmental Cell, 27, 452–461. 10.1016/j.devcel.2013.10.004 24286826

[pld3292-bib-0030] Kieber, J. J. , & Schaller, G. E. (2014). Cytokinins. The Arabidopsis Book, 12, e0168 10.1199/tab.0168 24465173 PMC3894907

[pld3292-bib-0031] Kim, H. J. , Park, J.‐H. , Kim, J. , Kim, J. J. , Hong, S. , Kim, J. , Kim, J. H. , Woo, H. R. , Hyeon, C. , Lim, P. O. et al (2018). Time‐evolving genetic networks reveal a NAC troika that negatively regulates leaf senescence in Arabidopsis. Proceedings of the National Academy of Sciences of the United States of America, 115, E4930–E4939. 10.1073/pnas.1721523115 PMC6003463 29735710

[pld3292-bib-0032] Kim, H. J. , Ryu, H. , Hong, S. H. , Woo, H. R. , Lim, P. O. , Lee, I. C. , Sheen, J. , Nam, H. G. , & Hwang, I. (2006). Cytokinin‐mediated control of leaf longevity by AHK3 through phosphorylation of ARR2 in Arabidopsis. Proceedings of the National Academy of Sciences of the United States of America, 103, 814–819. 10.1073/pnas.0505150103 16407152 PMC1334631

[pld3292-bib-0033] Kim, Y.‐S. , Sakuraba, Y. , Han, S.‐H. , Yoo, S.‐C. , & Paek, N.‐C. (2013). Mutation of the arabidopsis NAC016 transcription factor delays leaf senescence. Plant and Cell Physiology, 54, 1660–1672. 10.1093/pcp/pct113 23926065

[pld3292-bib-0034] Ko, D. , Kang, J. , Kiba, T. , Park, J. , Kojima, M. , Do, J. , Kim, K. Y. , Kwon, M. , Endler, A. , Song, W.‐Y. , Martinoia, E. , Sakakibara, H. , & Lee, Y. (2014). Arabidopsis ABCG14 is essential for the root‐to‐shoot translocation of cytokinin. Proceedings of the National Academy of Sciences of the United States of America, 111, 7150–7155. 10.1073/pnas.1321519111 24778257 PMC4024864

[pld3292-bib-0035] Kubiasová, K. , Montesinos, J. C. , Šamajová, O. , Nisler, J. , Mik, V. , Plíhalová, L. , Novák, O. , Marhavý, P. , Zalabák, D. , Berka, K. et al (2020). Cytokinin fluoroprobe reveals multiple sites of cytokinin perception at plasma membrane and endoplasmic reticulum. Nature communications, 11, 1‐11. 10.1038/s41467-020-17949-0 PMC7452891 32855390

[pld3292-bib-0036] Li, L. , Nelson, C. J. , Trösch, J. , Castleden, I. , Huang, S. , & Millar, A. H. (2017). Protein degradation rate in arabidopsis thaliana leaf growth and development. The Plant Cell, 29, 207–228. 28138016 10.1105/tpc.16.00768 PMC5354193

[pld3292-bib-0037] Li, Z. , Peng, J. , Wen, X. , & Guo, H. (2012). Gene network analysis and functional studies of senescence‐associated genes reveal novel regulators of arabidopsis leaf senescenceF. Journal of Integrative Plant Biology, 54, 526–539. 10.1111/j.1744-7909.2012.01136.x 22709441

[pld3292-bib-0038] Lim, P. O. , Kim, H. J. , & Gil Nam, H. (2007). Leaf SENESCENCE. Annual Review of Plant Biology, 58, 115–136. 10.1146/annurev.arplant.57.032905.105316 17177638

[pld3292-bib-0039] Lohman, K. N. , Gan, S. , John, M. C. , & Amasino, R. M. (1994). Molecular analysis of natural leaf senescence in Arabidopsis thaliana. Physiologia Plantarum, 92, 322–328. 10.1111/j.1399-3054.1994.tb05343.x

[pld3292-bib-0040] Love, M. I. , Huber, W. , & Anders, S. (2014). Moderated estimation of fold change and dispersion for RNA‐seq data with DESeq2. Genome Biology, 15, 550 10.1186/s13059-014-0550-8 25516281 PMC4302049

[pld3292-bib-0041] Meng, X. , Li, L. U. , De Clercq, I. , Narsai, R. , Xu, Y. , Hartmann, A. , Claros, D. L. , Custovic, E. , Lewsey, M. G. , Whelan, J. , & Berkowitz, O. (2019). ANAC017 coordinates organellar functions and stress responses by reprogramming retrograde signaling. Plant Physiology, 180, 634–653. 10.1104/pp.18.01603 30872424 PMC6501098

[pld3292-bib-0042] Miao, Y. , Laun, T. , Zimmermann, P. , & Zentgraf, U. (2004). Targets of the WRKY53 transcription factor and its role during leaf senescence in Arabidopsis. Plant Molecular Biology, 55, 853–867. 10.1007/s11103-005-2142-1 15604721

[pld3292-bib-0043] Mok, D. W. , & Mok M. C. (2001). Cytokinin metabolism and action. Annual Review of Plant Physiology and Plant Molecular Biology, 52, 89–118. 10.1146/annurev.arplant.52.1.89 11337393

[pld3292-bib-0044] Mok, M. C. , Martin, R. C. , & Mok, D. W. S. (2000). Cytokinins: biosynthesis, metabolism and perception. In Vitro Cellular & Developmental Biology ‐ Plant, 36, 102–107. 10.1007/s11627-000-0021-7

[pld3292-bib-0045] Nam, Y.‐J. , Tran, L.‐S.‐P. , Kojima, M. , Sakakibara, H. , Nishiyama, R. , & Shin, R. (2012). Regulatory roles of cytokinins and cytokinin signaling in response to potassium deficiency in arabidopsis. PLoS One, 7, e47797 10.1371/journal.pone.0047797 23112848 PMC3480408

[pld3292-bib-0046] Ng, S. , Ivanova, A. , Duncan, O. , Law, S. R. , Aken, O. V. , Clercq, I. D. , Wang, Y. , Carrie, C. , Xu, L. , Kmiec, B. et al (2013). A membrane‐bound NAC transcription factor, ANAC017, mediates mitochondrial retrograde signaling in arabidopsis. The Plant Cell, 25, 3450‐3471. 24045017 10.1105/tpc.113.113985 PMC3809543

[pld3292-bib-0047] Nishiyama, R. , Watanabe, Y. , Fujita, Y. , Le, D. T. , Kojima, M. , Werner, T. , Vankova, R. , Yamaguchi‐Shinozaki, K. , Shinozaki, K. , Kakimoto, T. , Sakakibara, H. , Schmülling, T. , & Tran, L.‐S. (2011). Analysis of cytokinin mutants and regulation of cytokinin metabolic genes reveals important regulatory roles of cytokinins in drought, salt and abscisic acid responses, and abscisic acid biosynthesis. The Plant Cell, 23, 2169–2183. 10.1105/tpc.111.087395 21719693 PMC3160038

[pld3292-bib-0048] Palni, L. M. S. , Palmer, M. V. , & Letham, D. S. (1984). The stability and biological activity of cytokinin metabolites in soybean callus tissue. Planta, 160, 242–249. 10.1007/BF00402861 24258507

[pld3292-bib-0049] Pernisova, M. , Grochova, M. , Konecny, T. , Plackova, L. , Harustiakova, D. , Kakimoto, T. , Heisler, M. G. , Novak, O. , & Hejatko, J. (2018). Cytokinin signalling regulates organ identity via the AHK4 receptor in Arabidopsis. Development, 145 10.1242/dev.163907 29967282

[pld3292-bib-0050] Pertea, M. , Kim, D. , Pertea, G. M. , Leek, J. T. , & Salzberg, S. L. (2016). Transcript‐level expression analysis of RNA‐seq experiments with HISAT, StringTie and Ballgown. Nature Protocols, 11, 1650–1667. 10.1038/nprot.2016.095 27560171 PMC5032908

[pld3292-bib-0051] Rashotte, A. M. , Carson, S. D. B. , To, J. P. C. , & Kieber, J. J. (2003). Expression profiling of cytokinin action in arabidopsis. Plant Physiology, 132, 1998–2011. 12913156 10.1104/pp.103.021436 PMC181285

[pld3292-bib-0052] Robatzek, S. , & Somssich, I. E. (2002). Targets of AtWRKY6 regulation during plant senescence and pathogen defense. Genes & Development, 16, 1139–1149. 12000796 10.1101/gad.222702 PMC186251

[pld3292-bib-0053] Romanov, G. A. , Lomin, S. N. , & Schmülling, T. (2006). Biochemical characteristics and ligand‐binding properties of Arabidopsis cytokinin receptor AHK3 compared to CRE1/AHK4 as revealed by a direct binding assay. Journal of Experimental Botany, 57, 4051–4058. 17075078 10.1093/jxb/erl179

[pld3292-bib-0054] Sakakibara, H. (2006). CYTOKININS: activity, biosynthesis, and Translocation. Annual Review of Plant Biology, 57, 431–449. 10.1146/annurev.arplant.57.032905.105231 16669769

[pld3292-bib-0055] Sakakibara, H. , Kasahara, H. , Ueda, N. , Kojima, M. , Takei, K. , Hishiyama, S. , Asami, T. , Okada, K. , Kamiya, Y. , Yamaya, T. et al (2005). Agrobacterium tumefaciens increases cytokinin production in plastids by modifying the biosynthetic pathway in the host plant. Proceedings of the National Academy of Sciences of the United States of America, 102, 9972–9977. 15998742 10.1073/pnas.0500793102 PMC1174980

[pld3292-bib-0056] Šimura, J. , Antoniadi, I. , Široká, J. , Tarkowská, D. , Strnad, M. , Ljung, K. , & Novák, O. (2018). Plant hormonomics: multiple phytohormone profiling by targeted metabolomics. Plant Physiology, 177, 476–489. 29703867 10.1104/pp.18.00293 PMC6001343

[pld3292-bib-0057] Singh, S. , Letham, D. S. , & Palni, L. M. S. (1992). Cytokinin biochemistry in relation to leaf senescence. VII. Endogenous cytokinin levels and exogenous applications of cytokinins in relation to sequential leaf senescence of tobacco. Physiologia Plantarum, 86, 388–397.

[pld3292-bib-0058] Singh, S. , Palni, L. M. S. , & Letham, D. S. (1992). Cytokinin biochemistry in relation to leaf senescence v. endogenous cytokinin levels and metabolism of zeatin riboside in leaf discs from green and senescent tobacco (Nicotiana rustica) leaves. Journal of Plant Physiology, 139, 279–283.

[pld3292-bib-0059] Skoog, F. , & Armstrong, D. (1970). Cytokinins. Annual Review of Plant Physiology, 21, 359–384.

[pld3292-bib-0060] Skoog, F. , & Miller, C. O. (1957). Chemical regulation of growth and organ formation in plant tissues cultured in vitro. Symposia of the Society for Experimental Biology, 54, 118–130. 13486467

[pld3292-bib-0061] Spíchal, L. , Rakova, N. Y. , Riefler, M. , Mizuno, T. , Romanov, G. A. , Strnad, M. , & Schmülling, T. (2004). Two cytokinin receptors of arabidopsis thaliana, CRE1/AHK4 and AHK3, differ in their ligand specificity in a bacterial assay. Plant and Cell Physiology, 45, 1299–1305. 10.1093/pcp/pch132 15509853

[pld3292-bib-0062] Sumanta, N. , Haque, C. I. , Nishika, J. , & Suprakash, R. (2014). Spectrophotometric analysis of chlorophylls and carotenoids from commonly grown fern species by using various extracting solvents. Research Journal of Chemical Sciences, 2231:606X.

[pld3292-bib-0063] Svačinová, J. , Novák, O. , Plačková, L. , Lenobel, R. , Holík, J. , Strnad, M. , & Doležal, K. (2012). A new approach for cytokinin isolation from Arabidopsis tissues using miniaturized purification: Pipette tip solid‐phase extraction. Plant Methods, 8, 17 10.1186/1746-4811-8-17 22594941 PMC3492005

[pld3292-bib-0064] Tian, T. , Liu, Y. , Yan, H. , You, Q. , Yi, X. , Du, Z. , Xu, W. , & Su, Z. (2017). agriGO v2.0: A GO analysis toolkit for the agricultural community, 2017 update. Nucleic Acids Research, 45, W122–W129. 28472432 10.1093/nar/gkx382 PMC5793732

[pld3292-bib-0065] To, J. P. , Haberer, G. , Ferreira, F. J. , Deruere, J. , Mason, M. G. , Schaller, G. E. , Alonso, J. M. , Ecker, J. R. , & Kieber, J. J. (2004). Type‐A Arabidopsis response regulators are partially redundant negative regulators of cytokinin signaling. The Plant Cell, 16, 658–671. 10.1105/tpc.018978 14973166 PMC385279

[pld3292-bib-0066] Tokunaga, H. , Kojima, M. , Kuroha, T. , Ishida, T. , Sugimoto, K. , Kiba, T. , & Sakakibara, H. (2012). Arabidopsis lonely guy (LOG) multiple mutants reveal a central role of the LOG‐dependent pathway in cytokinin activation. The Plant Journal, 69, 355–365. 10.1111/j.1365-313X.2011.04795.x 22059596

[pld3292-bib-0067] Ülker, B. , Shahid Mukhtar, M. , & Somssich, I. E. (2007). The WRKY70 transcription factor of Arabidopsis influences both the plant senescence and defense signaling pathways. Planta, 226, 125–137. 10.1007/s00425-006-0474-y 17310369

[pld3292-bib-0068] Vylíčilová, H. , Husičková, A. , Spíchal, L. , Srovnal, J. , Doležal, K. , Plíhal, O. , & Plíhalová, L. (2016). C2‐substituted aromatic cytokinin sugar conjugates delay the onset of senescence by maintaining the activity of the photosynthetic apparatus. Phytochemistry, 122, 22–33. 10.1016/j.phytochem.2015.12.001 26706318

[pld3292-bib-0069] Wang, J. , Ma, X.‐M. , Kojima, M. , Sakakibara, H. , & Hou, B.‐K. (2011). N‐Glucosyltransferase UGT76C2 is involved in cytokinin homeostasis and cytokinin response in arabidopsis thaliana. Plant and Cell Physiology, 52, 2200–2213. 10.1093/pcp/pcr152 22051886

[pld3292-bib-0070] Werner, T. , Nehnevajova, E. , Köllmer, I. , Novák, O. , Strnad, M. , Krämer, U. , & Schmülling, T. (2010). Root‐specific reduction of cytokinin causes enhanced root growth, drought tolerance, and leaf mineral enrichment in arabidopsis and tobacco. The Plant Cell, 22, 3905–3920. 21148816 10.1105/tpc.109.072694 PMC3027171

[pld3292-bib-0071] Woo, H. R. , Koo, H. J. , Kim, J. , Jeong, H. , Yang, J. O. , Lee, I. H. , Jun, J. H. , Choi, S. H. , Park, S. J. , Kang, B. , Kim, Y. W. , Phee, B.‐K. , Kim, J. H. , Seo, C. , Park, C. , Kim, S. C. , Park, S. , Lee, B. , Lee, S. , … Lim, P. O. (2016). Programming of plant leaf senescence with temporal and inter‐organellar coordination of transcriptome in arabidopsis. Plant Physiology, 171, 452–467. 10.1104/pp.15.01929 26966169 PMC4854694

[pld3292-bib-0072] Wulfetange, K. , Lomin, S. N. , Romanov, G. A. , Stolz, A. , Heyl, A. , & Schmulling, T. (2011). The cytokinin receptors of arabidopsis are located mainly to the endoplasmic reticulum. Plant Physiology, 156, 1808–1818. 10.1104/pp.111.180539 21709172 PMC3149959

[pld3292-bib-0073] Xiong, Y. , Contento, A. L. , & Bassham, D. C. (2005). AtATG18a is required for the formation of autophagosomes during nutrient stress and senescence in Arabidopsis thaliana. The Plant Journal, 42, 535–546. 10.1111/j.1365-313X.2005.02397.x 15860012

[pld3292-bib-0074] Yamada, H. , Suzuki, T. , Terada, K. , Takei, K. , Ishikawa, K. , Miwa, K. , Yamashino, T. , & Mizuno, T. (2001). The Arabidopsis AHK4 histidine kinase is a cytokinin‐binding receptor that transduces cytokinin signals across the membrane. Plant and Cell Physiology, 42, 1017–1023. 10.1093/pcp/pce127 11577198

[pld3292-bib-0075] Yang, S.‐D. , Seo, P. J. , Yoon, H.‐K. , & Park, C.‐M. (2011). The Arabidopsis NAC transcription factor VNI2 Integrates Abscisic Acid Signals into Leaf Senescence via the COR/RD Genes. The Plant Cell, 23, 2155–2168. 21673078 10.1105/tpc.111.084913 PMC3160032

[pld3292-bib-0076] Zhang, K. , Novak, O. , Wei, Z. , Gou, M. , Zhang, X. , Yu, Y. , Yang, H. , Cai, Y. , Strnad, M. , & Liu, C.‐J. (2014). Arabidopsis ABCG14 protein controls the acropetal translocation of root‐synthesized cytokinins. Nature Communications, 5, 1–12. 10.1038/ncomms4274 24513716

[pld3292-bib-0077] Zhang, Y. , Liu, Z. , Wang, X. , Wang, J. , Fan, K. , Li, Z. , & Lin, W. (2018). DELLA proteins negatively regulate dark‐induced senescence and chlorophyll degradation in Arabidopsis through interaction with the transcription factor WRKY6. Plant Cell Reports, 37, 981–992. 10.1007/s00299-018-2282-9 29574486

[pld3292-bib-0101] Zhou, X. , Jiang, Y. , & Yu, D. (2011). WRKY22 transcription factor mediates dark‐induced leaf senescence in Arabidopsis. Molecules and Cells, 31, 303–313. 21359674 10.1007/s10059-011-0047-1 PMC3933965

[pld3292-bib-0078] Zwack, P. J. , De Clercq, I. , Howton, T. C. , Hallmark, H. T. , Hurny, A. , Keshishian, E. A. , Parish, A. M. , Benkova, E. , Mukhtar, M. S. , Van Breusegem, F. , & Rashotte, A. M. (2016). Cytokinin response factor 6 represses cytokinin‐associated genes during oxidative stress. Plant Physiology, 172, 1249–1258. 10.1104/pp.16.00415 27550996 PMC5047073

[pld3292-bib-0079] Zwack, P. J. , Robinson, B. R. , Risley, M. G. , & Rashotte, A. M. (2013). Cytokinin response factor 6 negatively regulates leaf senescence and is induced in response to cytokinin and numerous abiotic stresses. Plant and Cell Physiology, 54, 971–981. 10.1093/pcp/pct049 23539244

[pld3292-bib-0080] Zwack, P. J. , Shi, X. , Robinson, B. R. , Gupta, S. , Compton, M. A. , Gerken, D. M. , Goertzen, L. R. , & Rashotte, A. M. (2012). Vascular expression and C‐terminal sequence divergence of cytokinin response factors in flowering plants. Plant and Cell Physiology, 53, 1683–1695. 10.1093/pcp/pcs110 22864451

