# Supplementary figures and images for: Cytokinin isopentenyladenine and its glucoside isopentenyladenine‐9G delay leaf senescence through activation of cytokinin‐associated genes
Source: Plant Direct. 2020 Dec 21;4(12):e00292. doi: 10.1002/pld3.292 (PMC7751127; doi:10.1002/pld3.292)

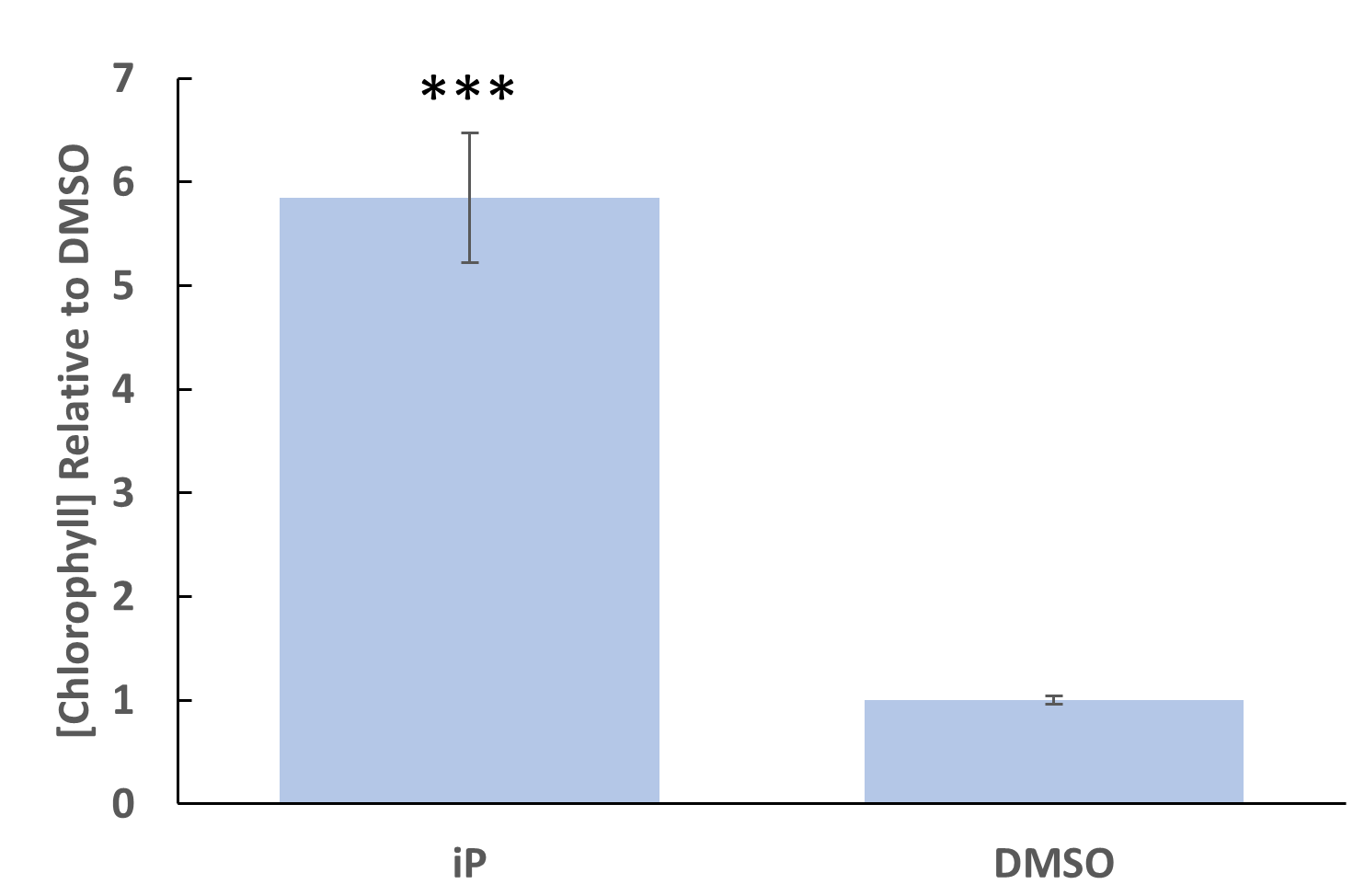

Supplement: Supplementary file 1 — Figure S1 [file PLD3-4-e00292-s001.tif]

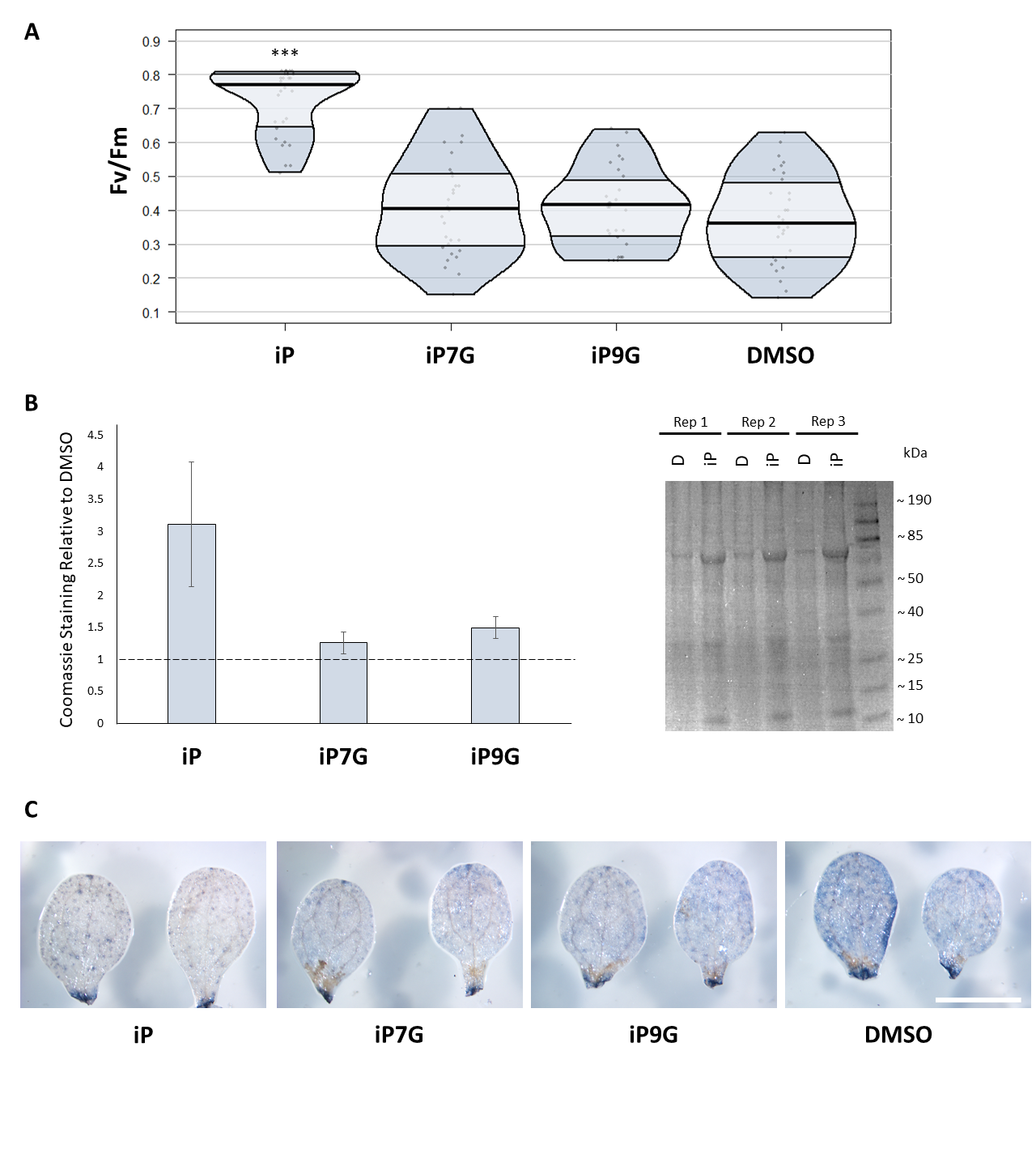

Supplement: Supplementary file 2 — Figure S2 [file PLD3-4-e00292-s002.tif]
