# Supplementary material for: Cytokinin isopentenyladenine and its glucoside isopentenyladenine‐9G delay leaf senescence through activation of cytokinin‐associated genes
Source: Plant Direct. 2020 Dec 21;4(12):e00292. doi: 10.1002/pld3.292 (PMC7751127; doi:10.1002/pld3.292)
Supplement: Supplementary file 3 — Table S1 [file PLD3-4-e00292-s003.docx]

Supplemental Table 1. Primer sequences for qPCR verification of RNA-sequencing. Primers are listed by Arabidopsis gene name abbreviation, direction of the primer F (Forward) and R (Reverse) as well as sequence.

Gene Primer Sequence

ARR5 F CTACTCGCAGCTAAAACGC

ARR5 R GCCGAAAGAATCAGGACA

ARR6 F TCGACGACAGTCACGTTGATCGTA

ARR6 R TCCAGTCATACCGGGCATTGAGTA

ARR7 F CTGGCATTGAGTAATCCGTCACTATC

ARR7 R TGACGACTGAGAAGGTGGAACTAGG

CKX4 F ACTATTGTCTCCAGGACAAGACA

CKX4 R TTCTGAGAGACCTATACCGCT

NAC17 F TGGTGCTCCGTTTCAAGAAGA

NAC17 R CCTCTCATCCACACGACGAG

SEN4 F CGTCGATGACACACCCATTAGAG

SEN4 R CATCGGCTTGTTCTTTGGAAAC

SAG12 F CGGATGTTGTTGGGCGTTTT

SAG12 R CCTTCGCAGCCAAAATCGTT

PDF1 F CCATTAGATCTTGTCTCTCTGCT

PDF1 R GACAAAACCCGTACCGAG
